# Host genetics and COVID-19 severity: increasing the accuracy of latest severity scores by Boolean quantum features

**DOI:** 10.3389/fgene.2024.1362469

**Published:** 2024-05-22

**Authors:** Gabriele Martelloni, Alessio Turchi, Chiara Fallerini, Andrea Degl’Innocenti, Margherita Baldassarri, Simona Olmi, Simone Furini, Alessandra Renieri, GEN-COVID Multicenter study, Francesca Mari

**Affiliations:** ^1^ Medical Genetics, University of Siena, Siena, Italy; ^2^ INAF Osservatorio Astrofisico di Arcetri, Florence, Italy; ^3^ Department of Medical Biotechnologies, Med Biotech Hub and Competence Center, University of Siena, Siena, Italy; ^4^ CNR-Consiglio Nazionale delle Ricerche—Istituto dei Sistemi Complessi, Sesto Fiorentino, Italy; ^5^ Department of Electrical, Electronic and Information Engineering “Guglielmo Marconi”, University of Bologna, Cesena, Italy; ^6^Genetica Medica, Azienda Ospedaliero-Universitaria Senese, Siena, Italy

**Keywords:** COVID-19, host genetics, integrated polygenic score, genetic algorithm, logistic regression, genetic science modeling

## Abstract

The impact of common and rare variants in COVID-19 host genetics has been widely studied. In particular, in Fallerini et al. (Human genetics, 2022, 141, 147–173), common and rare variants were used to define an interpretable machine learning model for predicting COVID-19 severity. First, variants were converted into sets of Boolean features, depending on the absence or the presence of variants in each gene. An ensemble of LASSO logistic regression models was used to identify the most informative Boolean features with respect to the genetic bases of severity. After that, the Boolean features, selected by these logistic models, were combined into an Integrated PolyGenic Score (IPGS), which offers a very simple description of the contribution of host genetics in COVID-19 severity.. IPGS leads to an accuracy of 55%–60% on different cohorts, and, after a logistic regression with both IPGS and age as inputs, it leads to an accuracy of 75%. The goal of this paper is to improve the previous results, using not only the most informative Boolean features with respect to the genetic bases of severity but also the information on host organs involved in the disease. In this study, we generalize the IPGS adding a statistical weight for each organ, through the transformation of Boolean features into “Boolean quantum features,” inspired by quantum mechanics. The organ coefficients were set via the application of the genetic algorithm PyGAD, and, after that, we defined two new integrated polygenic scores ( ${\text{IPGS}}_{ph}^{1}$ and ${\text{IPGS}}_{ph}^{2}$ ). By applying a logistic regression with both IPGS, ( ${\text{IPGS}}_{ph}^{2}$ (or indifferently ${\text{IPGS}}_{ph}^{1}$ ) and age as inputs, we reached an accuracy of 84%–86%, thus improving the results previously shown in Fallerini et al. (Human genetics, 2022, 141, 147–173) by a factor of 10%.

## 1 Introduction

COVID-19 disease, due to its rapid spreading worldwide, has led to the most severe pandemic since the deadly Spanish flu, which killed up to 100 million individuals in the past century. Most COVID-19-affected patients have mild symptoms, but approximately 20% of cases need hospitalization, with symptoms characteristic of severe to critical illness requiring very intensive help. Patients with severe illness are often older and/or have comorbidities (e.g., cardiovascular or chronic respiratory disease, diabetes, hypertension, and cancer). Moreover, the organ involvement turned out to be related to disease severity, even though the correlation is still under clarification Daga et al. (2021); Benetti et al. (2020a), while another factor that ended up being discriminant is gender, with men tending to have a more severe disease respect than women Wu et al. (2020). However, these factors do not fully explain the differences in severity and the fact that the immune responses to SARS-CoV-2 were variable, contributing in some cases to greater morbidity and mortality, due to the excessive inflammatory response Ballow and Haga (2021); Madabhavi et al. (2020).

It is now well-recognized that host genetic factors play a fundamental role in the COVID-19 clinical outcome. Recent advances in genome-wide associations have identified potential candidate genes in certain populations that may modify the host immune responses, leading to dysregulated host immunity. Different pathogenetic mechanisms can be involved as new genetic predisposing factors emerge, such as different immunogenicity/cytokine production capability, as well as receptor permissiveness to virus and antiviral defenses. Genetic defects of the type I interferon pathway are linked to a more clinically severe phenotype of COVID-19, and dysregulation of the adaptive immune system may play a role in the severity and complex clinical course of patients with COVID-19 Ballow and Haga (2021). However, with very few genetic factors identified until now, we are still very far from understanding the real relevance of host genetics. The better understanding of host genetic factors is fundamental to predict patients who are at a risk of severe disease and prevent and/or offer personalized and efficient treatments. Moreover, novel genetic discoveries could also inform therapeutic targets for drug repurposing, a pivotal example of which has been the discovery of homozygous deletions in the CCR5 gene conferring resistance to HIV-1 infection, which led to development of a drug that successfully made it through clinical trials Hütter et al. (2013).

Traditional methods for assessing the genetic bases of complex disorders include genome-wide association studies (GWASs) for common variants and burden tests for rare variants. GWASs focus mainly on common variants and are based on a comparison frequency of about 700,000 genomic single-nucleotide polymorphisms (SNPs) in cases/controls (mostly non-coding). The coverage of the coding SNPs is usually performed throughout imputed data, e.g., imputing 2 million SNPs from 700k SNPs by linkage disequilibrium. The method is based on multiple independent tests and has a high threshold for significance. Moreover, GWASs require sample sizes of ten-hundred thousand subjects COVID-19 Host Genetics Initiative (2021); Severe Covid-19 GWAS Group (2020); Kousathanas et al. (2021); Pairo-Castineira et al. (2021). On the other hand, the burden test is based on an aggregation of rare, protein-altering variants and a comparison between cases and controls. The reason behind the burden test is that grouping variants with a large effect size at a gene level might improve power. Like GWASs, the burden test method needs hundreds of thousands of participants for detection of statistically significant associations Kosmicki et al. (2021). These methods have been employed for many years but failed to fully unravel the complexity of human traits. Complex disorders such as COVID-19 are expected to be regulated by thousands of genes with different weights of contribution Marouli et al. (2017); Boyle et al. (2017). Indeed, in common genetic diseases such as cardiovascular or neurodegenerative disorders, the identified genetic markers were not sufficient for full use in clinical practice to predict and treat the disease.

To overcome these limitations, an interplay between host genetics, computational statistics, and dynamic system theory is necessary. Even though the scientific community has made a big effort to analyze the epidemic data made available by the Center for Systems Science and Engineering at Johns Hopkins University Dong et al. (2020), the applications of mean-field models able to predict the kinetics of the epidemic spreading Martelloni and Martelloni (2020a,b); Lai et al. (2020); Chen et al. (2020); Castorina et al. (2020); Fenga (2021); Fanelli and Piazza (2020); Agosto and Giudici (2020); Bialek et al., 2020; Lanteri et al. (2020) cannot help in identifying the gene variants that determine the risk of severity in order to understand the pathophysiological mechanisms responsible for severe disease in heterogeneous groups of patients. At the contrary, machine learning (ML) approaches offer an innovative tool for managing complex problems by significantly increasing our capacity to identify complex patterns of variations. Using data from the whole exome sequencing (WES), a first line of the ML method, i.e., a LASSO logistic regression, has been applied to extract some thousands of coding genetic features contributing to COVID-19 severity Picchiotti et al. (2021); Fallerini et al. (2022). Subsequent functional validation of extracted features demonstrated that, in each tested case, the association with severity has a biological basis and suggested hints for adjuvant treatment Benetti et al. (2020b); Fallerini et al. (2021b,a); Croci et al. (2022); Baldassarri et al. (2021b,a); Mantovani et al. (2022); Monticelli et al. (2021). Using the extracted features, Fallerini et al. (2022) build a severity score named the integrated polygenic score (IPGS), whose performances reached about 75% for both sensitivity and specificity. In this contribution, we want to improve the IPGS severity score performances, with the aim of increasing both metrics and the understanding of biomolecular mechanisms for personalized treatment using innovative ML methods. More in detail, we start from the same set of coding genetic features contributing to COVID-19 severity, already used in Picchiotti et al. (2021); Fallerini et al. (2022), to build two new severity scores that take into account the phenotype of the analyzed patients, i.e., the set of their observable characteristics or traits. In particular, we take into account, in the definition of the severity scores, the involvement of single organs in the development of the COVID-19 disease and the age of patients when they contract the virus. The contribution of single-organ involvement in developing severe COVID-19 disease and that of the gene frequency variants are estimated through an evolutionary algorithm usually implemented to generate high-quality optimization solutions. The severity scores we propose aim at reducing the enormous amount of data to treat and its complexity through a logistic regression, with the final goal of finding a correlation, for each patient, between the score itself and the severity of the disease registered according to the WHO COVID-19 Outcome Scale. In this way, the severity scores cannot be applied as predictive tools in clinical practice since they both require whole-exome sequencing done, the information on organ involvement, and a first screening through a LASSO logistic regression, which is done to extract the coding genetic features contributing to COVID-19 severity. However, they may help in investigating the relationship between gene variants with different frequencies and the development of severe COVID-19 disease.

The Methods section is devoted to the description of the implemented severity scores and the applied methods. Sec. 3 presents the performances of the new severity scores with respect to the IPGS, while a discussion on the presented results is reported in Section 4.

## 2 Methods

### 2.1 Data collection

Two different cohorts (from Italy and Sweden) contributed to this study, as described in detail in Supplementary Table S1. The Institutional Review Board approval was obtained for each study (see Institutional review board statement below). Information on the cohort demography is given in Table 1.

**TABLE 1:** Cohort demography information for male (upper table) and female (lower table) patient datasets.

Cohort	Number	Average age	Severe COVID-19 case	Mild COVID-19 case
Italy	1777	60.6	1,340	437
Sweden	88	59.4	88	0

Cohort	Number	Average age	Severe COVID-19 cases	Mild COVID-19 cases
Italy	1,222	60.1	715	507
Sweden	25	63.5	25	0

#### 2.1.1 Study participants and recruitment

In order to ensure a collection of samples that could be, as much as possible, comprehensive and representative of the Italian population, hospitals from across Italy, local healthcare units, and departments of preventive medicine have been involved in collecting samples and associated patient-level data for the GEN-COVID Multicenter Study^1^. The inclusion criteria for the study are as follows: PCR-positive SARS-CoV-2 infection, age ≥18 years, appropriately given informed consent that includes detailed information about the study, and maintaining the confidentiality of personal data. All subjects were positively diagnosed with SARS-CoV-2 and represented a wide range of disease severity, ranging from hospitalized patients with severe COVID-19 disease to asymptomatic individuals. The mean age of patients in the entire cohort is 60.9 years (range 18–99). The patients in the cohort are predominantly men (59.9%) with a mean age of 59.95 years (range 18–99); the mean age of women is 61.8 years (range 19–98). About 30.3% of patients in the cohort have no chronic conditions. The overall case-fatality rate is 2.5% with a mean age of 76.1 years [range 37–98]. Regarding ethnicity, the cohort is composed of 94.25% European, 2.51% Hispanic, 1.09% African, and 2.15% Asian patients. We included all the ethnicities in this study because the results do not depend on population structure-related confounding factors.

#### 2.1.2 Data collection and storage

The socio-demographic information included sex, age, and ethnicity. Information about family history, (pre-existing) chronic conditions, and SARS-CoV-2-related symptoms was collected through a detailed core clinical questionnaire where more than 160 clinical items have been listed (see Supplementary Table S2). Items concerning organ/system involvement (heart, liver, pancreas, kidney, and olfactory/gustatory and lymphoid systems) have been synthesized in a binary mode, where 1 means standard medical parameters indicating specific organ involvement (respiratory severity, taste/smell involvement, heart involvement, liver involvement, pancreas involvement, kidney involvement, lymphoid involvement, blood clotting, cytokine trigger, and a number of comorbidities like asthma, cancer, diabetes, dyslipidemia, hypertension, hypothyroidism, or obesity) and 0 means the absence of involvement of a certain organ/system. Peripheral blood samples were collected in ethylenediaminetetraacetic acid-containing tubes for all subjects, and aliquots of plasma are also available. Whenever possible, leukocytes were isolated from whole blood by density gradient centrifugation and stored in the dimethyl sulfoxide solution and frozen using liquid nitrogen. For the majority of the cohort, swab specimens are also available and stored at the reference hospitals. For more information on data collection and storage, refer to Benetti et al. (2020a); Daga et al. (2021).

#### 2.1.3 Phenotype definitions

COVID-19 severity has been assessed using a modified version of the WHO COVID-19 Outcome Scale (COVID-19 Therapeutic Trial Synopsis 2020); specifically six classification levels have been used to code for the severity: (5) death; (4) hospitalized, receiving invasive mechanical ventilation; (3) hospitalized, receiving continuous positive airway pressure or bilevel positive airway pressure ventilation; (2) hospitalized, receiving low-flow supplemental oxygen; (1) hospitalized, not receiving supplemental oxygen; and 0 not hospitalized. The number of patients present in each phenotype category of this six-level classification (termed GRADING_5_) is reported in Table 2. Through the application of the presented severity scores, this six-level classification will be reduced to three different classifications: i) a binary classification of patients into mild and severe cases (termed GRADING_2_), where a patient is considered severe if hospitalized and receiving any form of respiratory support (WHO severity grading equal to 4 or higher in six-point classification); ii) a three-level classification (termed GRADING_3_), where the patients are classified into non-hospitalized (WHO severity grading equal to 0 or 1), hospitalized and not receiving supplemental oxygen or receiving low-flow oxygen (WHO severity grading equal to 2 or 3), and patients with severe disease (WHO severity grading equal to 4 or higher); iii) a five-level classification (termed GRADING_4_), where the patients are classified into non-hospitalized (WHO severity grading equal to 0), hospitalized and not receiving supplemental oxygen (WHO severity grading equal to 1) or receiving low-flow oxygen (WHO severity grading equal to 2), hospitalized, receiving continuous positive airway pressure (WHO severity grading equal to 3), hospitalized, receiving invasive mechanical ventilation or dead (WHO severity grading equal to 4, 5).

**TABLE 2:** Numbers of patients present in each phenotype category for GRADING_5_.

GRADING_5_ level	Male	Female
0	201	298
1	227	184
2	589	367
3	465	220
4	252	74
5	122	78

#### 2.1.4 GEN-COVID cohort

Within the GEN-COVID Multicenter Study, biospecimens from more than 3,000 SARS-CoV-2-positive individuals were collected in the GEN-COVID Biobank (GCB) and used for identifying multi-organ involvement in COVID-19, defining genetic parameters for infection susceptibility within the population and mapping genetically COVID-19 severity and clinical complexity among patients. In particular, within the GEN-COVID Multicenter Study, about 3,000 patients were sequenced by whole-exome sequencing (WES) and partly (about 2,000) already included in the model described in Fallerini et al. (2022). WES with at least 97% coverage at 20x was performed using the Illumina NovaSeq 6000 System (Illumina, San Diego, CA, United States). Library preparation was performed using the Illumina Exome Panel (Illumina) according to the manufacturer’s protocol. Library enrichment was tested by qPCR, and the size distribution and concentration were determined using the Agilent Bioanalyzer 2100 (Agilent Technologies, Santa Clara, CA, United States). The NovaSeq 6000 System (Illumina) was used for DNA sequencing through 150 bp paired-end reads. Variant calling was performed according to the GATK4 (O’Connor and Auwera 2020) best practice guidelines, using BWA (Li and Durbin 2010) for mapping and ANNOVAR (Wang et al., 2010) for annotating.

#### 2.1.5 Swedish cohort

Whole-exome sequencing was performed using the Twist Bioscience exome capture probe and was sequenced on the Illumina NovaSeq 6000 platform. Data were then analyzed using the McGill Genome Center bioinformatics pipeline (https://doi.org/10.1093/gigascience/giz037) in accordance with GATK best practices.

### 2.2 Post-Mendelian paradigm for COVID-19 modelization

Fallerini et al. (2022) have developed an easily interpretable model that could be used to predict the severity of COVID-19 from host genetic data. Patients were considered severe when hospitalized and receiving any form of respiratory support. The focus on this target variable is motivated by the practical importance of rapidly identifying patients who are more likely to require oxygen support, in an effort to prevent further complications. The complexity of COVID-19 immediately suggests that both common and rare variants are expected to contribute to the likelihood of developing a severe form of the disease. However, the weight of contribution of common and rare variants to the severe phenotype is not expected to be the same. A single rare variant that impairs protein function might cause a severe phenotype by itself after viral infection, while this is not so probable for a common polymorphism, which is likely to have a less marked effect on protein functionality. These observations led to the definition of a score, named integrated polygenic risk score (IPGS), which includes data regarding the variants at different frequencies:

$$\begin{array}{c} IPGS=\left(n_{C}^{s}-n_{C}^{m}\right)+F_{LF}\left(n_{LF}^{s}-n_{LF}^{m}\right)\\ +F_{R}\left(n_{R}^{s}-n_{R}^{m}\right)+F_{UR}\left(n_{UR}^{s}-n_{UR}^{m}\right). \end{array}$$ (1)

In this equation, the *n* variables indicate the number of input features of the predictive model that promote the severe outcome (superscript s) or protect from a severe outcome (superscript m) and with genetic variants having minor allele frequency (MAF) ≥5% (common, subscript C), 1% ≤ MAF <5% (low-frequency, subscript LF), 0.1% ≤ MAF <1% (rare, subscript R), and MAF <0.1% (ultra-rare, subscript UR). The features promoting or preventing severity were identified by an ensemble of logistic models. The weighting factors *F*_*LF*_, *F*_*R*_, and *F*_*UR*_ model the different penetrant effects of low-frequency, rare, and ultra-rare variants, compared to common variants (for which the weighting factor has been chosen as 1). Thus, the four terms of Eq. 1 can be interpreted as the contributions of common, low-frequency, rare, and ultra-rare variants to a score that represents the genetic propensity of a patient to develop a severe form of COVID-19. In particular, note the difference in the sign between the severe and mild variants, which, respectively, represent a predisposing factor compared to a protection factor. The model including the IPGS exhibited an overall accuracy of 73% and precision of 78%, with a sensitivity and specificity of 72% and 75%, respectively, thus showing a statistically significant increase in the performances with respect to logistic models that adopt only age and sex as input features. However, in order to design prevention and treatment protocols in view of personalized medicine development, the predictability of the post-Mendelian paradigm for COVID-19 modelization should be further increased.

### 2.3 First phenotype-based IPGS ( ${\text{IPGS}}_{ph}^{1}$ )

To improve the ability of the IPGS to predict the severity of the disease, while keeping the linearity of the formula, we first apply vectorial formulation, where both the Boolean variables of the individual patients and the Boolean variables of the single variants are transformed into vectors with components 0 or 1. To each patient and each single variant is associated a vector, which has univocally defined non-zero components: the non-zero components of the patient vector *p*_*i*_ and the variants vector $v_{j}^{s,m}$ allow us to codify the situation of each patient who has a unique set of variants and a specific clinical condition when he/she has contracted the COVID-19 disease. Specifically, the clinical overview takes into account the involvement of the organs for each subject that are included in the matrix O, whose entries *O*_*ij*_ are 1 (0) in case the organ *j* is involved (noninvolved) in the disease development of patient *i*. The organ involvements are grouped into six categories (i.e., heart, liver, pancreas, kidney, olfactory/gustatory, and lymphoid systems), as mentioned in Sec. 2.1. Therefore, the matrix entries *O*_*ij*_ take into account, for each patient *i*, if one of the *j* = 6 categories are involved (*O*_*ij*_ = 1) or not involved (*O*_*ij*_ = 0). A scalar product between the vector of the single patient and the vectors of the genetic variants through the matrix of the organs univocally identifies the phenotypic characteristics of the patients, weighted by the variants. Finally, we release the condition that mild variants always protect from a severe outcome, thus being subtracted in Eq. 1, and we do not fix *a priori* the sign of the mild variants. Starting from a vectorial formulation of the severity score, we are now able to write down a severity score that includes not only the genetic features of the single patients but also the involvement of the organs in the disease development through the matrix of the organs *O*_*ij*_. The score index that encompasses the phenotypical characteristics of the patients is called ${\text{IPGS}}_{ph}^{1}$ , and it reads as

$$IPGS_{ph}^{1}=\underset{f}{\sum }F_{f}\left(\underset{s}{\sum }p_{i}O_{ij}v_{j}^{s}+{\left(-1\right)}^{\alpha }\underset{m}{\sum }p_{i}O_{ij}v_{j}^{m}\right),$$ (2)

where *F*_*f*_ is the coefficient representing the frequency of the variants, as shown in Eq. 1, and the subscript *f* identifies either common, low-frequency, rare, and ultra-rare variants. As introduced before, *p*_*i*_ represents the single patient vector, while $v_{j}^{s,m}$ represents the vector of severe or mild variants, where we can distinguish between severe and mild according to the superscript. Differently from Eq. 1, we do not fix the sign of the variants; therefore, in the sum over the mild variants, the sign remains a coefficient to be fitted through the parameter *α*. This results in having 17 more parameters to be fixed. Some examples of Eq. 2 are reported in Sec. 1 in the Supplementary Material; some case examples are specifically reported for different involved organs and different genetic features.

### 2.4 Second phenotype-based IPGS ( ${\text{IPGS}}_{ph}^{2}$ )

Inspired by quantum mechanics, we try to generalize the severity score presented in Eq. 2, explicitly introducing in the formula the age of each patient and leaving the possibility, thanks to the quantum mechanics formalism, to introduce into the new severity score expression more general phenotype definitions. For a brief introduction to the quantum mechanics formalism, see Sec. 2 of the Supplementary Material. Borrowing the formalism of quantum mechanics, we use the following elements to construct the second severity score ${\text{IPGS}}_{ph}^{2}$ :

• The patient is described in terms of a vector |*p* >, which represents a state in quantum mechanics and describes the condition of the single human being.
• The genetic variants are also expressed in terms of vectors $|v_{i}^{s}>$ , which represent a vector’s basis to calculate the expectation value of the physical observables.
• The organs can be considered the physical observable O, whose expectation value represents our quantum-like ${\text{IPGS}}_{ph}^{2}$ .
• The time related to the evolution operator represents the patient’s age.
• The mild or severe variants can be represented through a spin variable *s* which takes values 1/2 or −1/2.

In order to better clarify the role played by each single element in the severity score, we explicitly write down the values we assign to the new Boolean variables. More in detail, we can distinguish the state of the single *i* − *th* patient via assigning a sequence of values $p_{i}=\left(\begin{array}{c} 1\\ 0 \end{array}\right)$ or $p_{i}=\left(\begin{array}{c} 0\\ 1 \end{array}\right)$ . Since we are dealing with patients who have contracted COVID-19 but have different phenotypic characteristics (i.e., different organs involved in the disease course), the sequence of 2-dim vectors with 0 or 1 values is unique for each patient, and it allows selecting the right organ involvement when performing a scalar product. To gain a better insight into the construction of the severity score, we refer to Sec. 1 in the Supplementary Material. Similarly, the same concept is reported on the genetic variants: if the patient shows the *j* − *th* variant, the vector $v_{j}^{s}$ takes the values $v_{j}^{s}=\left(\begin{array}{c} 1\\ 0 \end{array}\right)$ ; otherwise, we assign $v_{j}^{s}=\left(\begin{array}{c} 0\\ 1 \end{array}\right)$ . We thus have constructed the quantum-like Boolean variables (or features), and we are ready to define the mathematical structure of ${\text{IPGS}}_{ph}^{2}$ :

$$IPGS_{ph}^{2}=\underset{v_{i}^{s}}{\sum }<p|e^{-ı\frac{H\left(t\right)}{\hbar }}\text{O}e^{ı\frac{H\left(t\right)}{\hbar }}|v_{i}^{s}>,$$

where *H*(*t*) is the Hamiltonian operator and $e^{-ı\frac{H\left(t\right)}{\hbar }}$ represents the time-evolution operator. To make the previous formula manageable, we perform some approximations, by inserting a completeness of the vectors of our base $\left|v_{j}^{s}><v_{j}^{s}\right|$ , which represents the genetic heritage of the human being:

$$IPGS_{ph}^{2}=\underset{v_{i}^{s}}{\sum }\underset{v_{j}^{s}}{\sum }<p\|v_{j}^{s}><v_{j}^{s}|e^{-ı\frac{H\left(t\right)}{\hbar }}\text{O}e^{ı\frac{H\left(t\right)}{\hbar }}|v_{i}^{s}>.$$

We can perform subsequent approximations along two different lines: either i) we suppose that the vectors of the variants are eigenvalues of the Hamiltonian *H*(*t*), or ii) we perform the infinite time limit of the system. In the first case, if we assume that *E*_*i*_ represents the eigenvalue of the Hamiltonian *H*(*t*) related to the precise state $|v_{i}^{s}>$ , we can approximate $e^{-ı\frac{E_{i}t}{\hbar }}≃1-\frac{E_{i}t}{\hbar }$ . *E*, corresponding in general to the total energy of the system, can be put in correlation with the comorbidity of the system human being. In this case, we obtain:

$$\begin{array}{ccc} IPGS_{ph}^{2} & = & \underset{v_{i}^{s}}{\sum }<p\|v_{i}^{s}>\underset{v_{j}^{s}}{\sum }E_{j}^{2}t^{2}<v_{j}^{s}|\text{O}|v_{i}^{s}>\\ & = & \left(IPGS\right)\underset{v_{j}^{s}}{\sum }E_{j}^{2}t^{2}<v_{j}^{s}|\text{O}|v_{j}^{s}>. \end{array}$$ (3)

In the latter case, the limit *t* → *∞* corresponds to the assumption that the patient has contracted COVID-19 and his/her status is characterized by a small number of variants that are only those relevant to the contraction/development of the disease. The small set of variants that are related to the disease and influence the clinic outcome of the patients can be called *variants of the saddle point* Caux (2016) and identified with $|v_{sp}^{s}>$ . In this case, the severity score reads as

$$\begin{array}{ccc} IPGS_{ph}^{2} & = & \underset{v_{i}^{s}}{\sum }<p\|v_{i}^{s}>\underset{v_{sp}^{s}}{\sum }<v_{sp}^{s}|\text{O}|v_{sp}^{s}>\\ & = & \left(IPGS\right)\underset{v_{sp}^{s}}{\sum }<v_{sp}^{s}|\text{O}|v_{sp}^{s}>. \end{array}$$ (4)

In both Eqs. 3, 4, the term $\sum_{v_{i}^{s}}<p\|v_{i}^{s}>$ is present, which represents the scalar product between the vector that identifies the patients’ clinical state and the vector taking into account the genetic variants. Thanks to the characterization of the single genetic variant in terms of the spin variable *s* (*s* = mild, severe), this scalar product constitutes the IPGS previously defined in Eq. 1. In other words, the scalar product $\sum_{v_{i}^{s}}<p\|v_{i}^{s}>$ is the overlap between the initial state, i.e., the state of the patient and the base of our system (the host genetics).

The severity score in Eq. 1 turns out to be corrected by a form factor that constitutes either the expectation value of the organs on the state of all genes, weighted with the age in Eq. 3, or the interplay between the variants of the genes, known to be associated to viral susceptibility and disease severity and patient status in Eq. 4. While the form factor present in Eq. 3 can be easily interpreted as the clinical status of the patient, where organs correlate with the genetic variants, the form factor in Eq. 4 has a more complex interpretation. Somehow, the vector $|v_{sp}^{s}>$ represents that the variants selected by LASSO regression in Fallerini et al. (2022) and Eq. 4 can be interpreted as the product between the scores previously defined in Eqs. 1, 2: $IPGS_{ph}^{2}≃\left(IPGS\right)\times \left(IPGS_{ph}^{1}\right)$ .

To summarize, although in the work of Fallerini et al. (2022) the presence or absence of a genetic variant is identified through a Boolean variable 1 or 0, essentially a bit of information, in the present work, in order to maintain the linearity of the problem, we define a quantum bit to identify the presence/absence of a variant. Therefore, we pass from a scalar variable (1 or 0) to a spin variable, thus allowing us to linearly increase the parameter space and improve the prediction of disease severity. Furthermore, being a multifactorial disease, when defining a score in terms of matrix variables, we are able to take age, sex, and organ involvements into account at the same time. In this respect, the mathematics of quantum mechanics seems the ideal environment to describe this type of problem. However, we are just using a quantum-like formalism when replacing Boolean variables with matrices, but we are not introducing any quantum feature in the machine learning algorithm. Irrespective of the fact that we have just taken inspiration from quantum mechanics, since in the previous definitions of ${\text{IPGS}}_{ph}^{2}$ , differently from quantum mechanical models, there is no real-time evolution and the vectors are fixed *a priori*, as well as the structure of the observables, using the quantum mechanics formalism helped us generalize the problem and build a severity score that, in principle, can be generalized to other diseases.

### 2.5 The genetic algorithm PyGAD

**FIGURE 1:**
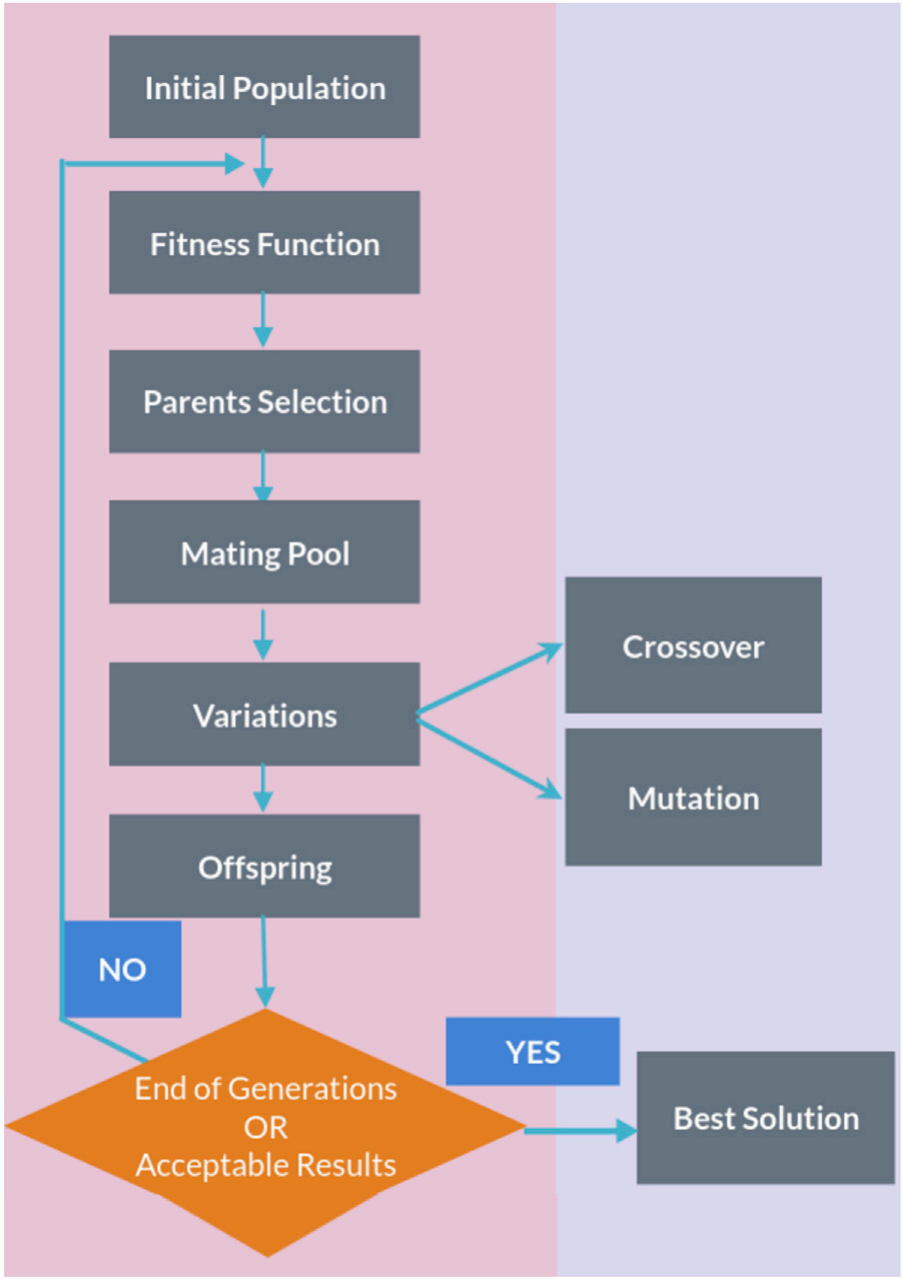
Flow chart of a genetic algorithm.

The genetic algorithm is a method for solving both constrained and unconstrained optimization problems that are inspired by natural selection, the process that drives biological evolution. In a genetic algorithm, we start with an initial population of chromosomes, which are possible solutions to a given problem. Those chromosomes consist of an array of genes whose values vary in a predefined range. The whole optimization problem is encoded into a fitness function, which receives a chromosome and returns a number that tells the fitness (or goodness) of the solution. The higher the fitness, the better the solution encoded in the chromosome. The genetic algorithm repeatedly modifies a population of individual solutions. At each step, the genetic algorithm selects individuals from the current population to be parents and uses them to produce the children for the next generation. At each iteration (generation), a number of good chromosomes are selected for breeding (parent selection). Parents are combined two-by-two (crossover) to generate new chromosomes (children). The children are finally mutated by (randomly) modifying part of their genes, allowing for completely new solutions to emerge. Over successive generations, the population “evolves” toward an optimal solution, as it is shown in the flow chart of a genetic algorithm (GA) in Figure 1.

**FIGURE 2:**
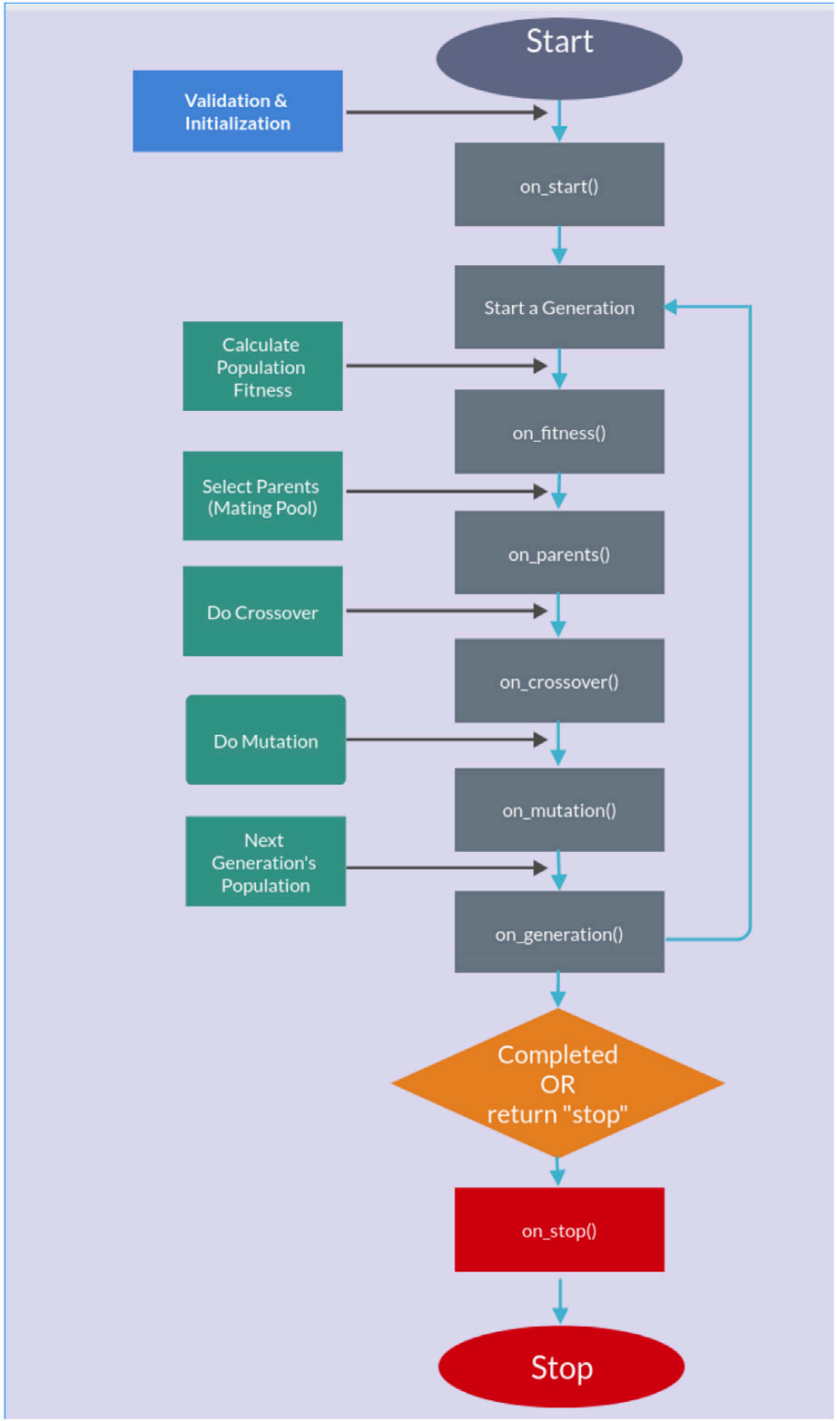
PyGAD lifecycle.

The genetic algorithm is usually applied to solve problems in which the objective function is discontinuous, non-differentiable, stochastic, or highly non-linear. Among the genetic algorithms, we find PyGAD, an open-source Python library Gad (2021), which supports a wide range of parameters to give the user control over everything in its cycle of operations (see Figure 2).

#### 2.5.1 Testing and training procedures

The dataset was randomly divided into a training set and a test set (50/50) for a total of 3,112 patients. In other words, half of the subjects (1,552) were used for training the model, and the remaining half (1,560) are used for testing the model. Patients are chosen randomly to be grouped into the training or the testing set, and the random sampling is varied across the study. Letting the algorithm perform training over a limited set of patients (50%) randomly chosen may potentially diminish the performances of the scores but allows for a more general solution, which is not limited to the particular set chosen for the training/test. The PyGAD algorithm was implemented with the following characteristics in order to converge to a stable solution:

• Number of solutions (i.e., chromosomes) within the population = 32.
• Number of generations 250–500.
• Number of solutions to be selected as parents = 8.
• Parent selection type = sss (for steady-state selection). In the sss case, only a few individuals are replaced at a time, meaning most of the individuals will carry out to the next generation.
• Number of parents to keep in the current population = 1.
• Crossover operation = single_point (for single-point crossover). All genes to the right of that point are swapped between the two parent chromosomes. This results in two offspring, each carrying some genetic information from both parents.
• Type of the mutation operation = random (for random mutation).
• The probability of selecting a gene for applying the mutation operation = 0.2 (for each gene in a solution, a random value with probability 20% is generated).

In most part of the developed training/testing tests, the number of generations able to guarantee a convergence of the solution is 250. We considered a converged solution to be one that has reached an asymptotic value within the duration of the test.

The training/testing procedure, for each severity score, was implemented separately on the male and female patient datasets. The whole procedure is made up of two parts, both used on the testing and training samples. In the first part, we let the genetic algorithm run over the training sample to fit the parameters of the severity scores in Eqs. 2, 3 that produce the best estimate of the *N*-level classification of patient severity (i.e., GRADING_*N*_ parameter). In particular, in this training process, the statistical weights for different organs are calculated without applying any constraint in the fitting process: we do not consider, for example, the possibility that the involvement of certain organs might lead to worse outcomes when compared to that by others. Then, ${\text{IPGS}}_{ph}^{1}$ ( ${\text{IPGS}}_{ph}^{2}$ ) is computed over the test sample by employing the fitted parameters. For each severity score and each dataset, the training and testing tests were repeated 10 times by varying the random sampling. Since the mutation process is random, this is done to ensure that we are able to get the best solution among a sufficient number of iterations. In the second part of the procedure, a multivariable logistic regression is fitted using ${\text{IPGS}}_{ph}^{1}$ ( ${\text{IPGS}}_{ph}^{2}$ ) computed according to the steps described above, together with other input parameters (age, IPGS, and sex), to predict the same GRADING_*N*_ parameter. The logistic model is first trained on the training sample and then tested on the test sample. The solutions that are shown in the following section are those corresponding to the best performances among the obtained results.

## 3 Results

The severity scores in Eqs. 2, 3 are used, together with the GRADING_*N*_ data, for training a model that predicts COVID-19 severity. In particular, the training procedure is devoted to fitting the parameters that are present in the severity score equations: 17 free parameters for Eqs. 2 and 18 free parameters for Eq. 3. Fitting the parameters will allow us to assess, for each patient, the level of severity of his/her COVID-19 infection, in terms of ${\text{IPGS}}_{ph}^{1}$ ( ${\text{IPGS}}_{ph}^{2}$ ). Since the final goal is to produce the *N*-level classification of patient severity, we have to further reduce the results obtainable from Eqs. 2, 3 in the N-level classification along the line of GRADING_*N*_.

To obtain the best possible fit, we have implemented the genetic algorithm PyGAD with the following step fitness function:

• We assign a reward 50 in case the obtained score value is ${\text{IPGS}}_{ph}^{1}$ ( ${\text{IPGS}}_{ph}^{2}$ ) = GRADING_*N*_ ± 0.5.
• We assign a reward 5 in case the obtained score value is ${\text{IPGS}}_{ph}^{1}$ ( ${\text{IPGS}}_{ph}^{2}$ ) = GRADING_*N*_ ± 1.
• We assign 0 otherwise.

The reward values are chosen without lack of generality: we have assigned a sufficiently big reward value when the algorithm is able to predict the right GRADING_*N*_ value, a small but non-zero reward value when the prediction is not too far from the right value and a 0 reward value when the prediction is completely wrong. Any other set of reward values chosen according to this principle, which ensures the convergence of the solution, will give comparable results.

### 3.1 GRADING_2_

First, we present the results related to GRADING_2_, where we have reduced the severity scores to a binary classification of patients into mild and severe cases, considering a patient severe (GRADING_2_ = 1) if hospitalized and receiving any form of respiratory support or healthy (GRADING_2_ = 0) in all the other cases. The number of patients present in each phenotype category for GRADING_2_ is reported in Table 3.

**TABLE 3:** Numbers of patients present in each phenotype category for GRADING_2_.

GRADING_2_ level	Male	Female
0	437	507
1	1,428	740

**FIGURE 3:**
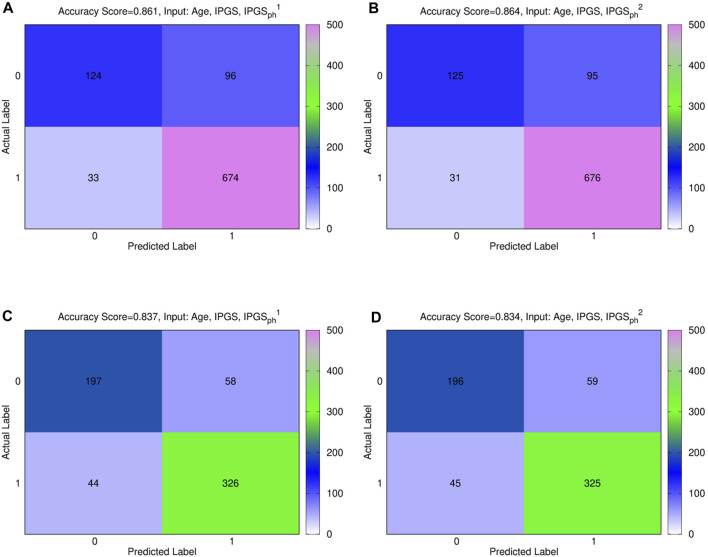
**(A)**, **(B)** confusion male matrix from logistic regression. **(A)** Input: age, IPGS, and ${\text{IPGS}}_{ph}^{1}$ . Accuracy = 86.1%. **(B)** Input: age, IPGS, and ${\text{IPGS}}_{ph}^{2}$ . Accuracy = 86.4%. **(C)**, **(D)** confusion female matrix from logistic regression. **(C)** Input: age, IPGS, and ${\text{IPGS}}_{ph}^{1}$ . Accuracy = 83.7%. **(D)** Input: age, IPGS, and ${\text{IPGS}}_{ph}^{2}$ . Accuracy = 83.4%.

Furthermore, a multivariable logistic regression was fitted using possible inputs ${\text{IPGS}}_{ph}^{1}$ and ${\text{IPGS}}_{ph}^{2}$ , alone or combined with IPGS, age, and sex. Figure 3 shows the confusion matrices, also known as error matrices Stehman (1997), for the male (panels (a) and (b)) and female (panels (c) and (d)) patient dataset, where the best fit is presented for both sets. The performances of the logistic regression increase when multiple predictor variables are used, instead of the single severity score ${\text{IPGS}}_{ph}^{1}$ ( ${\text{IPGS}}_{ph}^{2}$ ). In particular, the best fit is obtained, both for the male and female patient dataset, when using age, IPGS, and ${\text{IPGS}}_{ph}^{1}$ ( ${\text{IPGS}}_{ph}^{2}$ ) as inputs, while for male patients, the new severity score ${\text{IPGS}}_{ph}^{2}$ gives comparable accuracy results to ${\text{IPGS}}_{ph}^{1}$ , and for female patients, the logistic regression gives higher accuracy when giving age, IPGS, and ${\text{IPGS}}_{ph}^{1}$ as inputs, with respect to age, IPGS, and ${\text{IPGS}}_{ph}^{2}$ . However, the reached accuracy values are comparable for both severity scores; specifically, we reach an accuracy >86% for the male patient dataset and an accuracy >83% for the female patient dataset. Moreover, the confusion matrices indicate that we are able to predict grading 1 with a reasonably high successfully rate, while we have more difficulties in predicting grading 0. Most errors are done, both for the female and male patient datasets, when the actual score is 0, but we predict 1.

**FIGURE 4:**
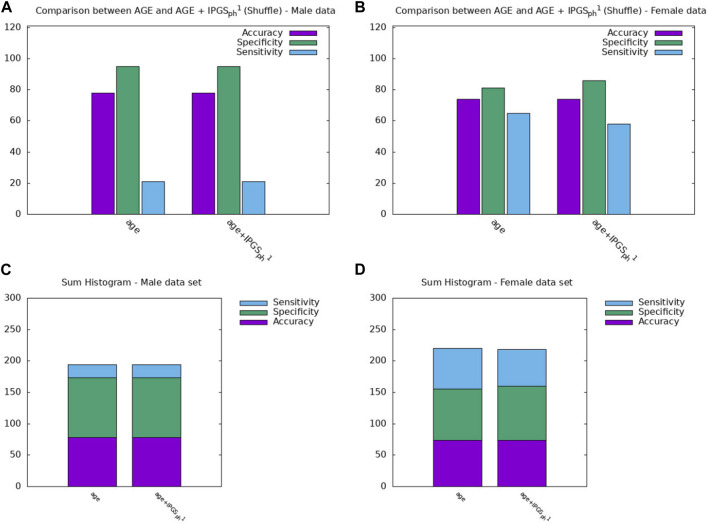
Comparison between the results obtained from a logistic regression with in input age or age+IPGS1 with shuffled variants for the **(A,C)** male sample and the **(B,D)** female sample.

In order to confirm the goodness of the results previously shown, we evaluate the increase in the performances of the severity score, as shown in Eqs. 2, 3, with respect to the performances of a model where the values of the ${\text{IPGS}}_{ph}^{1}$ feature have been shuffled. In other words, we recalculate ${\text{IPGS}}_{ph}^{1}$ by assigning to each patient a random distribution of variants instead of his/her genetic variants. To compare the results, we perform a logistic regression with the age as the input and (age + ${\text{IPGS}}_{ph}^{1}$ ) calculated with the shuffled variants (see Figure 4; and Table 4).The performances of the logistic regression with the age as the input and ${\text{IPGS}}_{ph}^{1}$ with shuffled variants are comparable with those obtained with only age as the input, thus confirming that the calculation of the severity score with shuffled variants does not add any information with respect to age. Moreover, in terms of accuracy, the score of the logistic regression shown in Figure 4m both for male (panel a) and female patients (panel b), is lower than the corresponding score presented in Figure 3. The accuracy for ${\text{IPGS}}_{ph}^{1}$ ( ${\text{IPGS}}_{ph}^{2}$ ) with the genetic variants is increased by a factor of 12% (10%) for the male (female) sample with respect to ${\text{IPGS}}_{ph}^{1}$ with shuffled variants when performing the logistic regression with age, IPGS, and ${\text{IPGS}}_{ph}^{1}$ as inputs (age, IPGS, and ${\text{IPGS}}_{ph}^{2}$ ). This means that the contribution of genetic variants to the information is fundamental in our analysis, in addition to the age factor that seems to be dominant in determining the severity of the disease.

**TABLE 4:** Accuracy, sensitivity, and specificity scores resulting from the logistic regression for GRADING_2_. The table above (below) shows the results obtained from the male (female) patient dataset.

Input variable	Accuracy	Specificity	Sensitivity
age	0.78	0.95	0.21
age+ ${\text{IPGS}}_{ph}^{1}$ (shuffle)	0.78	0.95	0.21

Input variables	Accuracy	Specificity	Sensitivity
age	0.74	0.81	0.65
age+ ${\text{IPGS}}_{ph}^{1}$ (Shuffle)	0.74	0.86	0.58

**FIGURE 5:**
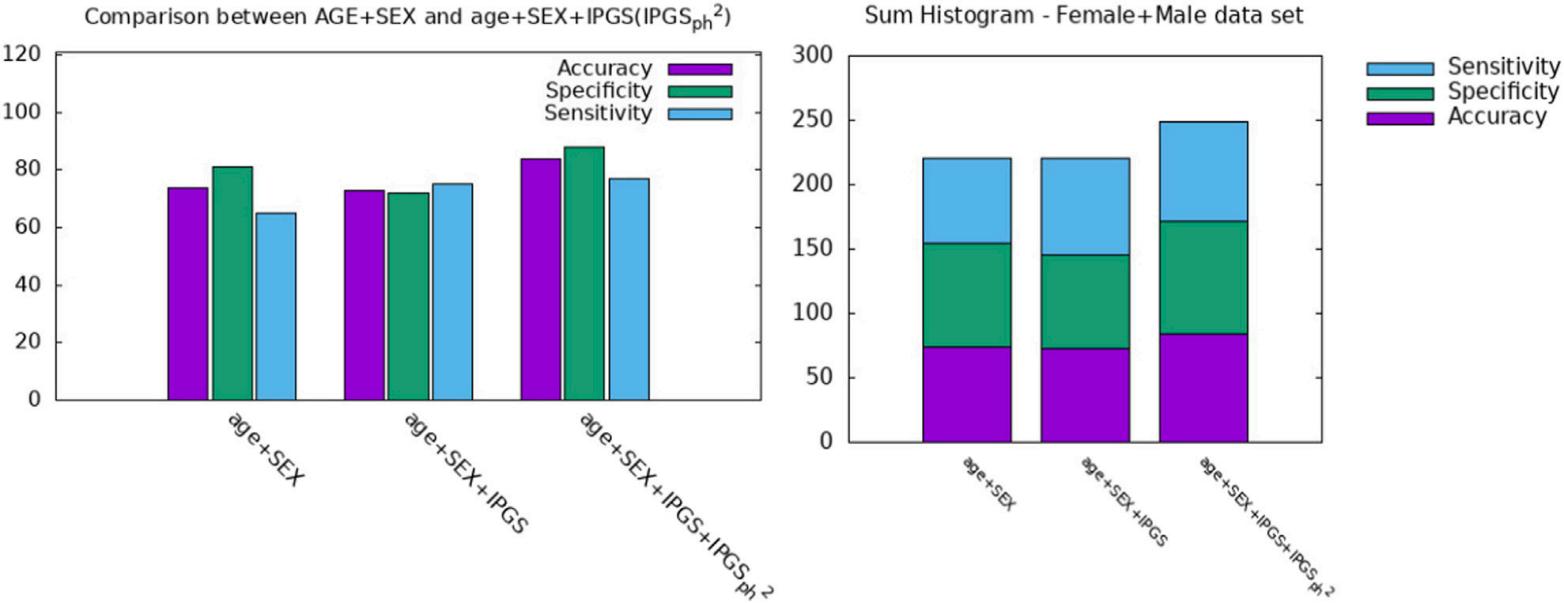
Comparison between AGE + SEX, AGE + SEX + IPGS, and AGE + SEX + IPGS+ ${\text{IPGS}}_{ph}^{2}$ as inputs at the logistic regression on the total (female + male) dataset.

**FIGURE 6:**
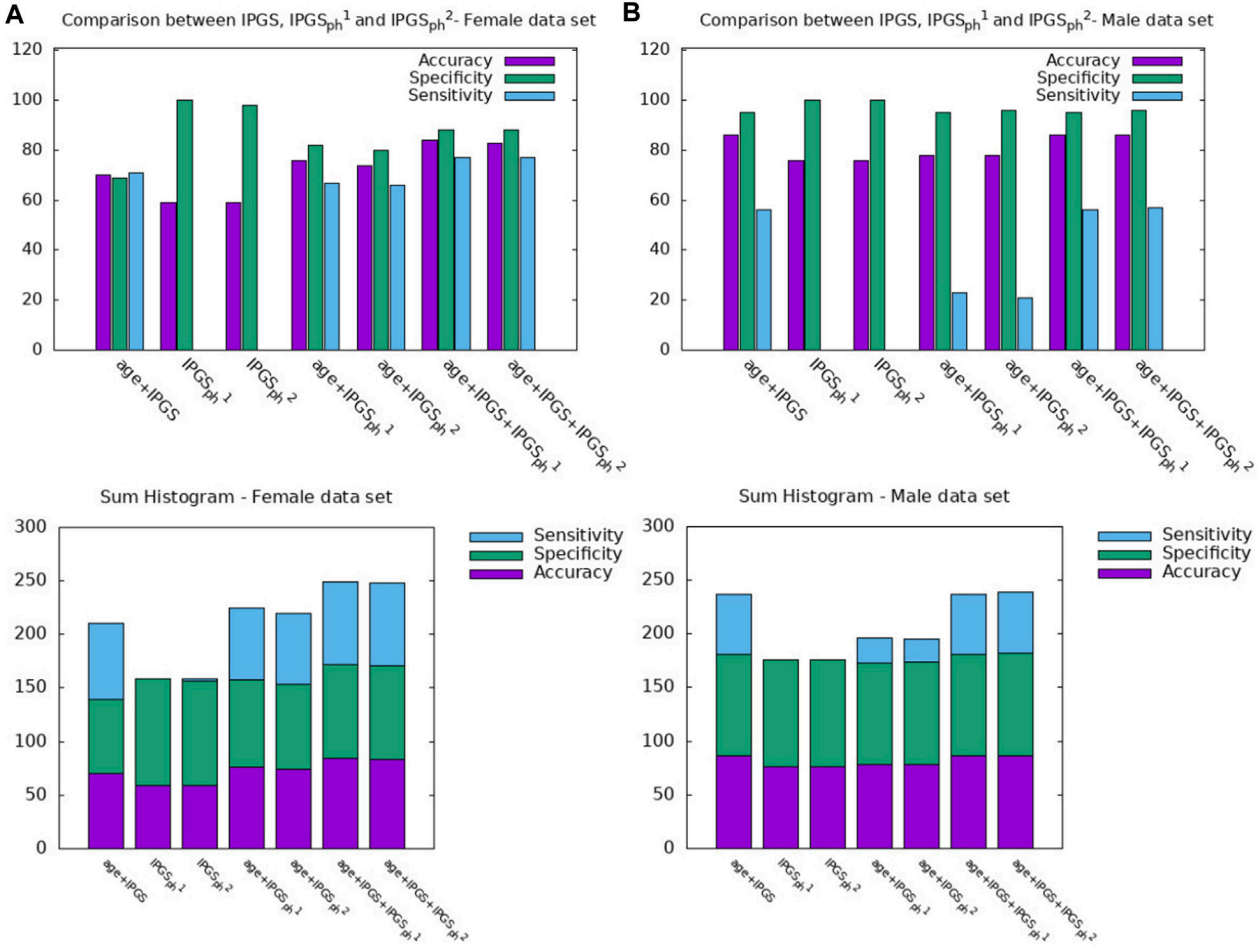
Comparison between IPGS, ${\text{IPGS}}_{ph}^{1}$ , and ${\text{IPGS}}_{ph}^{2}$ for the female **(A)** and male **(B)** samples.

In order to further investigate the role played by age and other factors that seem to be discriminant, i.e., sex, in comparison with the new severity scores presented here, we report a comparison between the performances of the logistic regression when the predictor variables in the input are (age + sex), (age + sex + IPGS), or (age + sex + IPGS + ${\text{IPGS}}_{ph}^{2}$ ) (see Figure 5 and Table 5 for an overview). Here, we report just the results for ${\text{IPGS}}_{ph}^{2}$ , but comparable results are obtained when fitting the logistic regression with (age + sex) or (age + sex + IPGS + ${\text{IPGS}}_{ph}^{1}$ ) as the input. Considering both the role played by the genetic variants through the IPGS and the phenotypic information on the patients through ${\text{IPGS}}_{ph}^{1}$ ( ${\text{IPGS}}_{ph}^{2}$ ), we observe an improvement in sensitivity, specificity, and accuracy scores with respect to the case where only the information on age and sex is used as the input for logistic regression. This confirms the initial hypotheses that comorbidities, age, and sex are important to determine the disease severity, but these factors do not fully explain the differences in severity. More in detail, when comparing the results of the logistic regression with (age and IPGS), (age and ${\text{IPGS}}_{ph}^{1}$ ), (age and ${\text{IPGS}}_{ph}^{2}$ ), (age, IPGS, and ${\text{IPGS}}_{ph}^{1}$ ), or (age, IPGS, and ${\text{IPGS}}_{ph}^{2}$ ) as inputs for the female (Figure 6 (a)) and male (Figure 6 (b)) patient datasets, we observe that the best performances are obtained when using age, IPGS, and ${\text{IPGS}}_{ph}^{1}$ ( ${\text{IPGS}}_{ph}^{2}$ ) as input data. The numerical values corresponding to the histogram representation in Figure 6 are reported in Tables 6, 7.

**TABLE 5:** Accuracy, sensitivity, and specificity scores resulting from the logistic regression on both female and male patient datasets for GRADING_2_.

Input variable	Accuracy	Specificity	Sensitivity
Age + SEX	0.74	0.81	0.65
Age + SEX + IPGS	0.70	0.69	0.71
Age + SEX + IPGS + ${\text{IPGS}}_{ph}^{2}$	0.84	0.88	0.77

**TABLE 6:** Accuracy, sensitivity, and specificity scores resulting from the logistic regression on the female patient dataset for GRADING_2_.

Input variable	Accuracy	Specificity	Sensitivity
Age + IPGS	0.70	0.69	0.71
${\text{IPGS}}_{ph}^{1}$	0.59	1	0.0039
${\text{IPGS}}_{ph}^{2}$	0.59	0.98	0.016
Age+ ${\text{IPGS}}_{ph}^{1}$	0.76	0.82	0.67
Age+ ${\text{IPGS}}_{ph}^{2}$	0.74	0.80	0.66
Age + IPGS + ${\text{IPGS}}_{ph}^{1}$	0.84	0.88	0.77
Age + IPGS + ${\text{IPGS}}_{ph}^{2}$	0.83	0.88	0.77

**TABLE 7:** Accuracy, sensitivity, and specificity scores resulting from the logistic regression on the male patient dataset for GRADING_2_.

Input variables	Accuracy	Specificity	Sensitivity
Age + IPGS	0.82	0.92	0.37
${\text{IPGS}}_{ph}^{1}$	0.76	1	0
${\text{IPGS}}_{ph}^{2}$	0.76	1	0
Age+ ${\text{IPGS}}_{ph}^{1}$	0.78	0.95	0.23
Age+ ${\text{IPGS}}_{ph}^{2}$	0.78	0.96	0.21
Age + IPGS + ${\text{IPGS}}_{ph}^{1}$	0.86	0.95	0.56
Age + IPGS + ${\text{IPGS}}_{ph}^{2}$	0.86	0.96	0.57

Since the applied method is stochastic, for completeness, we also report the accuracy, sensitivity, and specificity scores averaged over the different training and testing tests that we specify for the case where the logistic regression has inputs (age, IPGS, and ${\text{IPGS}}_{ph}^{1}$ ). For the female patient dataset, the average scores are accuracy = 0.83 ± 0.05, specificity = 0.880 ± 0.002, and sensitivity = 0.768 ± 0.004, while for the male patient dataset, the average scores are accuracy = 0.859 ± 0.003, specificity = 0.952 ± 0.001, and sensitivity = 0.556 ± 0.008. Comparable results can be obtained for the logistic regression with the inputs (age, IPGS, and ${\text{IPGS}}_{ph}^{2}$ ), thus confirming the stability of the analysis.

Therefore age, genetic variants, and organ involvements seem to all concur and contribute to the amount of information necessary to reach good levels of sensitivity and accuracy scores ( >80% in all cases). Moreover, taking into account the organs involved during the disease, each with its statistical weight, leads to an improvement in the score of 10% compared to the previous work (corresponding to the case where age, sex, and IPGS are used as input variables), in terms of forecast accuracy. Poor performances are obtained when calculating the sensitivity, especially for the male patient dataset. Since the performances are poorer when the input variables to the logistic regression are smaller, especially when age or ${\text{IPGS}}_{ph}^{1}$ ( ${\text{IPGS}}_{ph}^{2}$ ) are the only input variables, we expect a failure of logistic regression due to the small ratio between the information provided and the complexity of the parameter space in which it has to operate. It is worth noticing that the calculation of the logistic regression with input age + IPGS + ${\text{IPGS}}_{ph}^{1}$ (age + IPGS + ${\text{IPGS}}_{ph}^{2}$ ) as the input is done over a dataset where half of the subjects are used for training and the remaining subjects are used for testing the model, and, in both cases, subjects are randomly chosen. This approach is based on the assumption that the IPGS is the additional independent information provided to the algorithm. However, since the testing set used in this work is partially overlapping with the samples used in Fallerini et al. (2022) to define the IPGS score, this assumption is not entirely valid. When testing the logistic regression only on the testing set used in Fallerini et al. (2022), we obtain the accuracy = 0.70, sensitivity = 0.71, and specificity = 0.70 for the female patient dataset, while for the male patient dataset, we obtain the accuracy = 0.83, sensitivity = 0.38, and specificity = 0.92, thus resulting in performances that are comparable (higher) for the female (male) patient dataset with respect to the logistic regression with age + IPGS as the input. In the same way, when calculating the logistic regression with age + SEX + IPGS + ${\text{IPGS}}_{ph}^{2}$ as the input on the testing set used in Fallerini et al. (2022), the results reported in Table 5 vary to accuracy = 0.70, sensitivity = 0.71, and specificity = 0.69, in line with the results obtained for age + SEX + IPGS. The lower performances in this case are due to the fact that the ratio in the training/testing set in Fallerini et al. (2022) is 90/10; therefore, we are implementing the logistic regression on a much smaller dataset than before, not compensating with an equivalent increase in the training set.

Finally, we spend some words on the comparison between ${\text{IPGS}}_{ph}^{1}$ and ${\text{IPGS}}_{ph}^{2}$ (see Figure 6 and Tables 6, 7). Analyzing the performances of the logistic regression with in input the severity scores ${\text{IPGS}}_{ph}^{1}$ and ${\text{IPGS}}_{ph}^{2}$ taken separately, we note that the proposed severity score models are substantially equivalent. The small differences in terms of accuracy scores within the same sample are due to the genetic algorithm procedure: when different minima, but close in the parameter space, are reached, the algorithm cannot easily escape, and we accept the proposed solution as the asymptotic one. However, it is worth noticing that a relevant difference remains when comparing the results obtained on the male and female patient datasets. For female subjects, the single scores reach an accuracy of about 59%, while for the male sample, we obtain an excellent 76% accuracy, contrary to what we have seen in Figure 6, where the logistic regression with other variables as inputs (such as age and IPGS) allows us to obtain similar results for the male and female data samples. We can speculate that different results in the two data samples are due to the differences in the genetic pool between male and female subjects since the total number of genes contributing to COVID-19 clinical variability was 4,260 in male and 4,360 in female subjects, 75% of which were in common. Therefore, the non-common set of genes (25%) may be determinant in giving different results. Another hypothesis is related to the fact that male subjects are more prone to have a more severe disease compared to female subjects; therefore, we have more phenotypic data for males and more male patients analyzed (1865 male with respect to 1,247 female subjects): more specific information in this case means better training and higher performances in the testing procedure. Discrepancies in the model performance between genders have been already found and discussed in Fallerini et al. (2022) on the same female and male patient datasets, while they are quite known in the literature Mukherjee and Pahan (2021); Jin et al. (2020); Gebhard et al. (2020); O’Brien et al. (2020).

However, it is clear that the information on the organ involvement is independent of the chosen severity score model and that the genetic PyGAD algorithm works very well in highlighting this aspect. The proposed severity scores perform comparably within the same sample data because they are both able to convey all the relevant information from the clinical data collection, even though they are derived from different principles and are functionally different.

### 3.2 GRADING_3_

In this second part of the section, we present the results related to GRADING_3_, where we have reduced the severity scores to a three-level classification of patients into non-hospitalized (GRADING_3_ = 0), hospitalized but not receiving supplemental oxygen or receiving low-flow oxygen (GRADING_3_ = 1), and patients with severe disease (GRADING_3_ = 2). In this last case, patients are considered to manifest a severe disease when they are hospitalized and receiving intensive or invasive respiratory support or are dead. The number of patients present in each phenotype category for GRADING_3_ is reported in Table 8.

**TABLE 8:** Number of patients present in each phenotype category for GRADING_3_.

GRADING_3_ level	Male	Female
0	210	323
1	816	551
2	839	373

**FIGURE 7:**
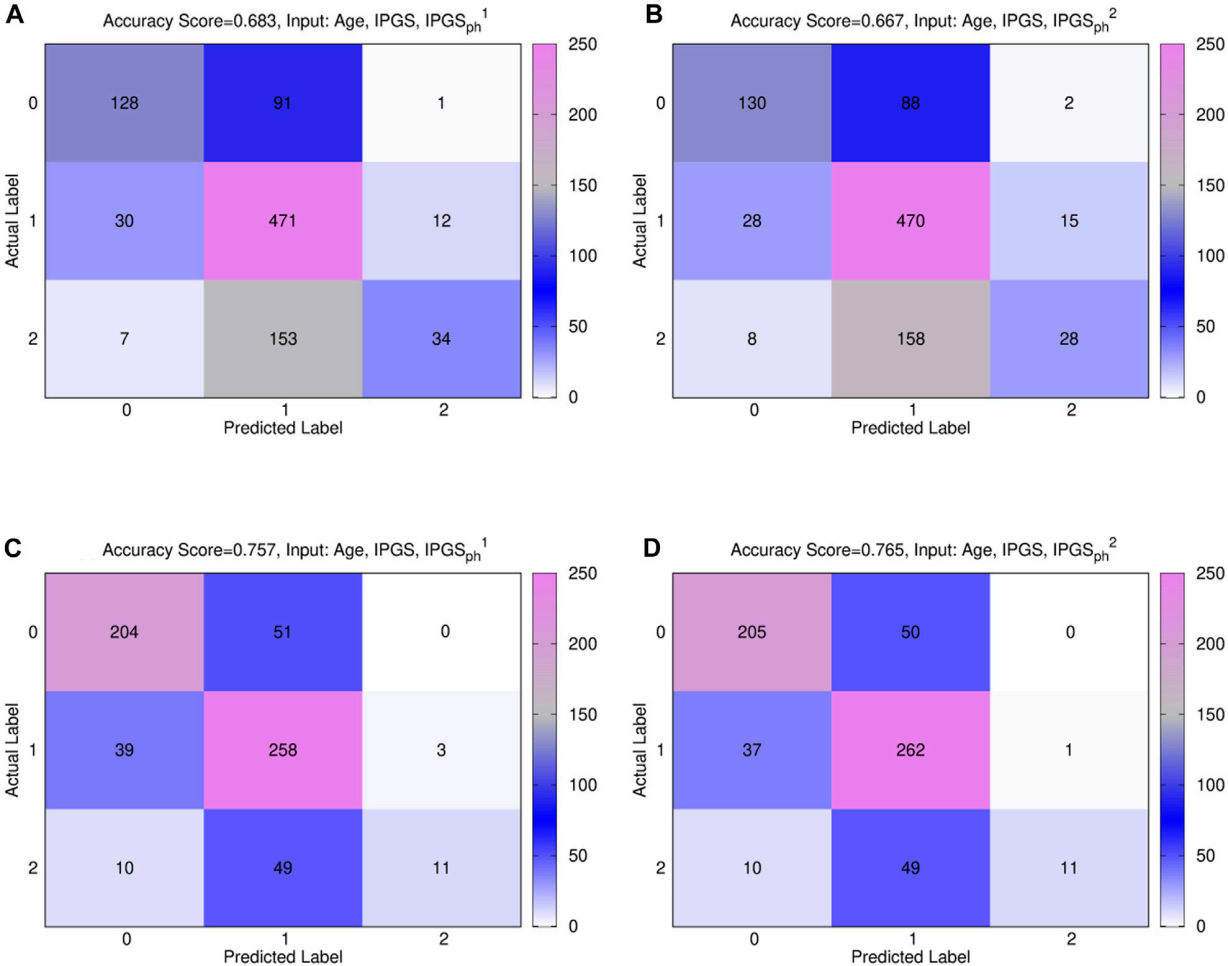
Comparison between ${\text{IPGS}}_{ph}^{1}$ and ${\text{IPGS}}_{ph}^{2}$ . Confusion matrices obtained from the logistic regression of the single scores with age and IPGS for the male (panels **(A)**, **(B)**) and female (panels **(C)**, **(D)** datasets. Panels **(A)** and **(C)** show results for ${\text{IPGS}}_{ph}^{1}$ and **(B)** and **(D)** for ${\text{IPGS}}_{ph}^{2}$

Figure 7 shows the confusion matrices for the male (panels (a) and (b)) and female (panels (c) and (d)) patient datasets, where the best fit is presented for both sets. The results are relative to a logistic regression with multiple predictor variables used as inputs: age, IPGS, and ${\text{IPGS}}_{ph}^{1}$ for panels (a) and (c); age, IPGS, and ${\text{IPGS}}_{ph}^{2}$ for panels (b) and (d). Within the same dataset, the performances of the severity scores are comparable, while, comparing between the two datasets, the accuracy experienced on the female sample is higher than the one on the male sample, irrespectively of the chosen severity score.

In general, the accuracy reached in each case for GRADING_3_ is lower than the accuracy reached for GRADING_2_, as shown in Figure 3, due to binning limitations. If we look in detail at the confusion matrices shown in Figure 7, it turns out that the biggest errors are done in two cases: i) when we have to predict 0 and we predict 1; ii) when we have to predict 2 and we predict 1. Probably the information that we have on the clinical framework of each patient is not optimized for distinguishing between low and severe disease, thus explaining why the GRADING_2_ was performing better since the algorithm was not required to distinguish between low and severe disease for GRADING_2_, but all the hospitalized patients were treated in the same way. A general comparison of the performances of the logistic regression on GRADING_3_ is shown in Tables 9, 10 for the female and male patient datasets, respectively. Analogously to the results obtained for GRADING_2_, the performances are enhanced when calculating the logistic regression on age, IPGS, and ${\text{IPGS}}_{ph}^{1}$ ( ${\text{IPGS}}_{ph}^{2}$ ), with respect to the calculation on age and IPGS only. To ensure the stability of the analysis, we report, as previously done, the accuracy, sensitivity, and specificity scores averaged over the different training and testing tests. Specifically, we consider the case where the logistic regression has the input (age, IPGS, and ${\text{IPGS}}_{ph}^{1}$ ). For the female patient dataset, the average scores are accuracy = 0.750 ± 0.008, specificity = 0.67 ± 0.09, and sensitivity = 0.813 ± 0.0005, while for the male patient dataset, the average scores are accuracy = 0.676 ± 0.007, specificity = 0.64 ± 0.10, and sensitivity = 0.808 ± 0.002. Comparable results can be obtained for the logistic regression with the input (age, IPGS, and ${\text{IPGS}}_{ph}^{2}$ ). Moreover, when testing the logistic regression only on the testing set used in Fallerini et al. (2022) to ensure a completely independent testing set, we obtain the accuracy = 0.594, sensitivity = 0.692, and specificity = 0.522 for the female dataset , while for the male dataset, we obtain the accuracy = 0.584, sensitivity = 0.840, and specificity = 0.824, thus resulting in performances that are comparable for both datasets with respect to the logistic regression with the input (age + IPG).

**TABLE 9:** Accuracy, sensitivity, and specificity scores resulting from the logistic regression on the female patient dataset for GRADING_3_. The calculation of precision and sensitivity is done by applying the sklearn.metrics module Pedregosa et al. (2011); Kramer and Kramer (2016) in Python. The algorithm calculates the metrics for each label and finds their average scores, weighted by the number of true instances for each label.

Input variable	Accuracy	Sensitivity	Specificity
Age + IPGS	0.596	0.695	0.582
Age + ${\text{IPGS}}_{ph}^{1}$	0.656	0.740	0.653
Age + ${\text{IPGS}}_{ph}^{2}$	0.654	0.738	0.646
Age + IPGS + ${\text{IPGS}}_{ph}^{1}$	0.757	0.813	0.761
Age + IPGS + ${\text{IPGS}}_{ph}^{2}$	0.765	0.827	0.765

**TABLE 10:** Accuracy, sensitivity, and specificity scores resulting from the logistic regression on the male patient dataset for GRADING_3_. The calculation of precision and sensitivity is done by applying the sklearn.metrics module Pedregosa et al. (2011); Kramer and Kramer (2016) in Python. The algorithm calculates the metrics for each label and finds their average scores, weighted by the number of true instances for each label.

Input variable	Accuracy	Sensitivity	Specificity
Age + IPGS	0.582	0.838	0.255
Age + ${\text{IPGS}}_{ph}^{1}$	0.594	0.839	0.285
Age + ${\text{IPGS}}_{ph}^{2}$	0.589	0.870	0.239
Age + IPGS + ${\text{IPGS}}_{ph}^{1}$	0.683	0.810	0.538
Age + IPGS + ${\text{IPGS}}_{ph}^{2}$	0.677	0.814	0.529

### 3.3 GRADING_4_

We finally present the results related to GRADING_4_, where we have applied the WHO severity grading in five points to classify the patients, merging the classes (4) and (5). The number of patients present in each phenotype category for GRADING_4_ is reported in Table 11.

**TABLE 11:** Number of patients present in each phenotype category for GRADING_4_.

GRADING_4_ level	Male	Female
0	210	323
1	227	184
2	589	367
3	465	220
4	374	153

**FIGURE 8:**
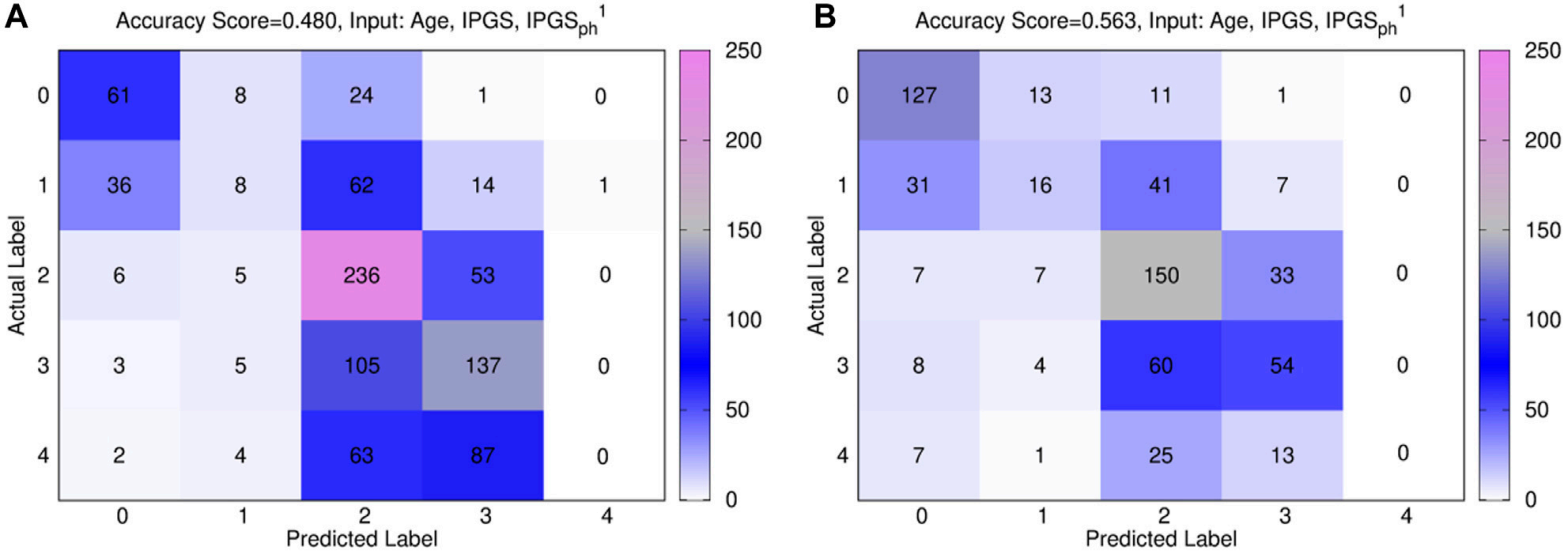
Confusion matrices obtained from the logistic regression of the single scores with age, IPGS, and ${\text{IPGS}}_{ph}^{1}$ for the male (panels **(A)**) and female (panels **(B)**) patient datasets.

Figure 8 shows the confusion matrices for the male (panels (a)) and female (panels (b)) patient datasets, where the best fit is presented for both sets. The results are relative to a logistic regression with multiple predictor variables are used as inputs: age, IPGS, and ${\text{IPGS}}_{ph}^{1}$ for both panels. Since ${\text{IPGS}}_{ph}^{1}$ and ${\text{IPGS}}_{ph}^{2}$ have shown to give comparable results, here we report the results just for ${\text{IPGS}}_{ph}^{1}$ . Moreover, a general comparison of the performances of the logistic regression on GRADING_4_ is shown in Tables 12, 13 for the female and patient male datasets, respectively.

**TABLE 12:** Accuracy, specificity, and sensitivity scores resulting from a logistic regression on the female patient dataset for GRADING_4_. The calculation of specificity and sensitivity is done by applying the sklearn.metrics module Pedregosa et al. (2011); Kramer and Kramer (2016) in Python. The algorithm calculates the metrics for each label and finds their average scores, weighted by the number of true instances for each label.

Input variable	Accuracy	Sensitivity	Specificity
Age + IPGS	0.332	0.639	0.356
Age + ${\text{IPGS}}_{ph}^{1}$	0.445	0.785	0.441
Age + IPGS + ${\text{IPGS}}_{ph}^{1}$	0.563	0.709	0.724

**TABLE 13:** Accuracy, specificity, and sensitivity scores resulting from a logistic regression on the male patient dataset for GRADING_4_. The calculation of specificity and sensitivity is done by applying the sklearn.metrics module Pedregosa et al. (2011); Kramer and Kramer (2016) in Python. The algorithm calculates the metrics for each label and finds their average scores, weighted by the number of true instances for each label.

Input variable	Accuracy	Sensitivity	Specificity
Age + IPGS	0.306	0.572	0.286
Age + ${\text{IPGS}}_{ph}^{1}$	0.341	0.871	0.138
Age + IPGS + ${\text{IPGS}}_{ph}^{1}$	0.480	0.681	0.585

In general, the accuracy reached in each case for GRADING_4_ is lower than those reached for both GRADING_2_ and GRADING_3_. If we look in detail at the confusion matrices presented in Figure 8, the biggest errors are related to the false-positive values detected for classes 3 and 4. While the algorithm seems to identify quite well the classes 0, 1, and 2, more difficulties are encountered when it comes to distinguishing between the class levels relative to severe disease. Finally, if we compare the results of the logistic regression performed with (age and IPGS) as inputs with those obtained with inputs (age, IPGS, and ${\text{IPGS}}_{ph}^{1}$ ), we observe a slight increase in the performances when considering two severity scores at the same time (in line with what is shown for GRADING_3_ and GRADING_2_). Analogously, a slight increase in the performances is observed if we compare the results of the logistic regression with (age and ${\text{IPGS}}_{ph}^{1}$ ) as inputs with respect to the analogous case with age and IPGS as inputs (as seen also for GRADING_2_ and GRADING_3_). Finally, to ensure the stability of the analysis we report, as previously done, the accuracy, sensitivity, and specificity scores are averaged over different training and testing tests. Specifically, we consider the case where the logistic regression has (age, IPGS, and ${\text{IPGS}}_{ph}^{1}$ ) as inputs. For the female patient dataset, the average scores are accuracy = 0.560 ± 0.003, specificity = 0.720 ± 0.004, and sensitivity = 0.708 ± 0.001, while for the male patient dataset, the average scores are accuracy = 0.476 ± 0.004, specificity = 0.582 ± 0.003, and sensitivity = 0.673 ± 0.008. Moreover, when testing the logistic regression only on the testing set used in Fallerini et al. (2022) to ensure a completely independent testing set, we obtain for the female data set an accuracy = 0.326, sensitivity = 0.611, and specificity = 0.349, while for the male dataset, we obtain accuracy = 0.302, sensitivity = 0.640, and specificity = 0.280, thus resulting in performances that are comparable for both datasets with respect to the logistic regression with (age and IPGS) as inputs.

## 4 Conclusion

In this article, we have presented two severity scores that, starting from the integrated polygenic score (IPGS) introduced in Fallerini et al. (2022), integrate the phenotype of the analyzed patients in order to improve the accuracy, sensitivity, and specificity performances registered by the IPGS. The performances of the proposed methods, based on a combination of clinical and genetic information, are higher than the performances of methods based on genetic information alone, as testified by the results of the logistic regression with age+ ${\text{IPGS}}_{ph}^{1}$ ( ${\text{IPGS}}_{ph}^{2}$ ) as the input with respect to the results with the input age + IPGS. Moreover, we propose to combine the information given by the IPGS, with the information supported by the new severity scores ${\text{IPGS}}_{ph}^{1}$ ( ${\text{IPGS}}_{ph}^{2}$ ) when performing the logistic regression, as we have observed that, in general, the best performances are obtained when using age, IPGS, and ${\text{IPGS}}_{ph}^{1}$ ( ${\text{IPGS}}_{ph}^{2}$ ) as input data. We believe that there is still the possibility to improve the performances of the algorithm either choosing the patients to belong to the testing/training set not completely random or including some constraints on the calculation of the statistical weights determining the organ involvements. In the first case, it would be worth choosing the patients proportionally to the number of cases present in each phenotype level of GRADING_*N*_ to avoid that some categories with a low number of cases are underrepresented in the training phase. In the latter case, it would be worth considering that the involvement of certain organs might lead to worse outcomes (e.g., kidney), with respect to others (e.g., olfactory/gustatory system).

However, since both our scores include information about organ involvements, which are available only in the course of the viral infection, these scores cannot be used as predictive tools in the general population, thus resulting as the main limitation of the study. Another limitation of this study is that the estimated performances are likely an overestimation of the predictive performances in a completely independent cohort, i.e., one that is not used to identify the genetic features to be used in the IPGS score. However, this limitation does not affect the main result of the study, which is the comparison in performances between IPGS and the new proposed scores. Irrespectively of the scores’ inability to make predictions on phenotype information since the information on the clinical history of each patient is needed to train the model, it is possible to profit from severity scores when investigating the role played by genetic variants in influencing the host response. The coefficients *F*_*f*_ representing the frequency of the variants as well as the sign *α* of the mild variants remain coefficients to be fitted through the ML algorithm. In this way, we could explore and test different possibilities, such as different *F*_*f*_ coefficients weighting the contribution of different variants (while in Fallerini et al. (2022), *F*_*f*_ was assumed to be all equal to 1), or different signs for the mild variants, thus releasing the hypothesis that mild variants are always protective.

The development of a tool able to predict, prior to viral infection, if one will be severely affected would have a tremendous impact on the social life and world economy, improving our capability of treatment and thus reducing mortality. In this view, the COVID-19 disease represents an ideal scenario for developing methods that could be used for other complex disorders since, compared to other complex disorders, in COVID-19, the environmental trigger is well-known (e.g., SARS-CoV-2 infection).

## Data Availability

Publicly available datasets were analyzed in this study. Sequencing data have been deposited in CINECA through http://www.nig.cineca.it/, specifically, http://nigdb.ext.cineca.it/, in the COVID-19 section through https://www.nig.cineca.it/?page_id=25. There are no restrictions on data access. Only registration is needed. A section dedicated to COVID-19 samples has been created within the NIG database (http://nigdb.ext.cineca.it/) that provides variant frequencies as a free tool for both clinicians and researchers. The GEN-COVID Biobank (GCB), a collection of biospecimens from patients affected by COVID-19, and the associated GEN-COVID Patient Registry (GCPR) were established and maintained at the University of Siena using the infrastructure of an already well-established biobank (est. 1998) (http://www.biobank.unisi.it/). The data and samples housed in the GEN-COVID Patient Registry and the GEN-COVID Biobank are available for consultation. For consultation, you may contact the last author, AR (e-mail: alessandra.renieri@unisi.it). The data from high-density (700k) SNP genotyping are also generated on the same cohort and shared with international collaborations, including the COVID-19 Host Genetics Initiative (https://www.covid19hg.org/) and with GoFAIR VODAN [COVID-19 Host Genetics Initiative. The COVID-19 Host Genetics Initiative, a global initiative to elucidate the role of host genetic factors in susceptibility and severity of the SARS-CoV-2 virus pandemic. Eur J Hum Genet. 2020;28:715–8]

## Footnotes

1 The GEN-COVID Multicenter Study includes a network of 22 Italian hospitals, 13 from Northern Italy, 5 from Central Italy, and 4 from Southern Italy. It also includes local healthcare units and departments of preventative medicine (https://sites.google.com/dbm.unisi.it/gen-covid).

## GEN-COVID Multicenter study (https://sites.google.com/dbm.unisi.it/gen-covid)

Francesca Mari, Sergio Daga, Ilaria Meloni, Mirella Bruttini, Susanna Croci, Mirjam Lista, Debora Maffeo, Elena Pasquinelli, Giulia Brunelli, Kristina Zguro, Viola Bianca Serio, Enrica Antolini, Simona Letizia Basso, Samantha Minetto, Giulia Rollo, Martina Rozza, Angela Rina, Rossella Tita, Maria Antonietta Mencarelli, Caterina Lo Rizzo, Anna Maria Pinto, Francesca Ariani, Francesca Montagnani, Mario Tumbarello, Ilaria Rancan, Massimiliano Fabbiani, Francesco Bianchi, and Nicola Picchiotti (Medical Genetics, University of Siena, Siena, Italy; Med Biotech Hub and Competence Center, Department of Medical Biotechnologies, University of Siena, Siena, Italy; Genetica Medica, Azienda Ospedaliero-Universitaria Senese, Siena, Italy; Department of Medical Sciences, Infectious and Tropical Diseases Unit, Azienda Ospedaliera-Universitaria Senese, Siena, Italy); Elena Bargagli, Laura Bergantini, Miriana d’Alessandro, Paolo Cameli, and David Bennett (Unit of Respiratory Diseases and Lung Transplantation, Department of Internal and Specialist Medicine, University of Siena, Siena, Italy); Federico Anedda, Simona Marcantonio, Sabino Scolletta, and Federico Franchi (Department of Emergency and Urgency, Medicine, Surgery, and Neurosciences, Unit of Intensive Care Medicine, Siena University Hospital, Siena, Italy); Maria Antonietta Mazzei and Susanna Guerrini (Department of Medical, Surgical, and Neurosciences and Radiological Sciences, Unit of Diagnostic Imaging, University of Siena, Italy); Edoardo Conticini, Luca Cantarini, and Bruno Frediani (Rheumatology Unit, Department of Medicine, Surgery and Neurosciences, University of Siena, Policlinico Le Scotte, Italy); Danilo Tacconi, Chiara Spertilli Raffaelli, and Arianna Emiliozzi (Department of Specialized and Internal Medicine, Infectious Diseases Unit, San Donato Hospital Arezzo, Italy); Marco Feri, Alice Donati (Department of Emergency, Anesthesia Unit, San Donato Hospital, Arezzo, Italy); Raffaele Scala and Luca Guidelli (Department of Specialized and Internal Medicine, Pneumology Unit and UTIP, San Donato Hospital, Arezzo, Italy); Genni Spargi and Marta Corridi (Department of Emergency, Anesthesia Unit, Misericordia Hospital, Grosseto, Italy); Cesira Nencioni and Leonardo Croci (Department of Specialized and Internal Medicine, Infectious Diseases Unit, Misericordia Hospital, Grosseto, Italy); Gian Piero Caldarelli (Clinical Chemical Analysis Laboratory, Misericordia Hospital, Grosseto, Italy); Davide Romani, Paolo Piacentini, Maria Bandini, Elena Desanctis, and Silvia Cappelli (Dipartimento di Prevenzione, Azienda Usl Toscana sud est, Italy); Anna Canaccini, Agnese Verzuri, Valentina Anemoli, and Manola Pisani (Dipartimento Tecnico-Scientifico Territoriale, Azienda Usl Toscana sud est, Italy); Agostino Ognibene, Maria Lorubbio, and Alessandro Pancrazzi (UOC Laboratorio Analisi Chimico Cliniche, Arezzo, Italy); Massimo Vaghi (Chirurgia vascolare, Ospedale Maggiore di Crema, Italy); Antonella D’Arminio Monforte, and Federica Gaia Miraglia (Department of Health Sciences, Clinic of Infectious Diseases, ASST Santi Paolo e Carlo, University of Milan, Italy); Mario U. Mondelli, Stefania Mantovani, Raffaele Bruno, Marco Vecchia, and Marcello Maffezzoni (Division of Clinical Immunology—Infectious Diseases, Department of Medicine, Fondazione IRCCS Policlinico San Matteo, Pavia, Italy; Department of Internal Medicine and Therapeutics, University of Pavia, Pavia, Italy); Enrico Martinelli (Department of Respiratory Diseases, Azienda Ospedaliera di Cremona, Cremona, Italy); Massimo Girardis, Stefano Busani, and Sophie Venturelli (Department of Anesthesia and Intensive Care, University of Modena and Reggio Emilia, Modena, Italy); Andrea Cossarizza (Department of Medical and Surgical Sciences for Children and Adults, University of Modena and Reggio Emilia, Modena, Italy); Andrea Antinori and Alessandra Vergori (HIV/AIDS Department, National Institute for Infectious Diseases, IRCCS, Lazzaro Spallanzani, Rome, Italy); Stefano Rusconi, Matteo Siano, Arianna Gabrieli, and Agostino Riva (III Infectious Diseases Unit, ASST-FBF-Sacco, Milan, Italy; Department of Biomedical and Clinical Sciences Luigi Sacco, University of Milan, Milan, Italy); Daniela Francisci, Elisabetta Schiaroli, and Carlo Pallotto (Infectious Diseases Clinic, “Santa Maria della Misericordia” Hospital, University of Perugia, Perugia, Italy); Saverio Giuseppe Parisi, and Monica Basso (Department of Molecular Medicine, University of Padova, Italy); Sandro Panese and Stefano Baratti (Clinical Infectious Diseases, Mestre Hospital, Venezia, Italy); Pier Giorgio Scotton, Francesca Andretta, and Mario Giobbia (Department of Infectious Diseases, Treviso Hospital, Local Health Unit 2 Marca Trevigiana, Treviso, Italy); Renzo Scaggiante and Francesca Gatti (Infectious Diseases Clinic, ULSS1, Belluno, Italy); Francesco Castelli, Eugenia Quiros-Roldan, and Melania Degli Antoni (Department of Infectious and Tropical Diseases, University of Brescia and ASST Spedali Civili Hospital, Brescia, Italy); Isabella Zanella (Department of Molecular and Translational Medicine, University of Brescia, Italy; Clinical Chemistry Laboratory, Cytogenetics and Molecular Genetics Section, Diagnostic Department, ASST Spedali Civili di Brescia, Italy); Matteo della Monica and Carmelo Piscopo (Medical Genetics and Laboratory of Medical Genetics Unit, A.O.R.N. “Antonio Cardarelli,” Naples, Italy); Mario Capasso, Roberta Russo, Immacolata Andolfo, Achille Iolascon, and Giuseppe Merla (Department of Molecular Medicine and Medical Biotechnology, University of Naples Federico II, Naples, Italy; CEINGE Biotecnologie Avanzate, Naples, Italy); Giuseppe Fiorentino (Unit of Respiratory Physiopathology, AORN dei Colli, Monaldi Hospital, Naples, Italy); Massimo Carella and Marco Castori (Division of Medical Genetics, Fondazione IRCCS Casa Sollievo della Sofferenza Hospital, San Giovanni Rotondo, Italy); Giuseppe Merla and Gabriella Maria Squeo (Laboratory of Regulatory and Functional Genomics, Fondazione IRCCS Casa Sollievo della Sofferenza); Filippo Aucella (Department of Medical Sciences, Fondazione IRCCS Casa Sollievo della Sofferenza Hospital, San Giovanni Rotondo, Italy); Pamela Raggi and Rita Perna (Clinical Trial Office, Fondazione IRCCS Casa Sollievo della Sofferenza Hospital, San Giovanni Rotondo, Italy); Matteo Bassetti and Antonio Di Biagio (Department of Health Sciences, University of Genova, Genova, Italy; Infectious Diseases Clinic, Policlinico San Martino Hospital, IRCCS for Cancer Research Genova, Italy); Maurizio Sanguinetti, Luca Masucci, and Alessandra Guarnaccia (Microbiology, Fondazione Policlinico Universitario Agostino Gemelli IRCCS, Catholic University of Medicine, Rome, Italy; Department of Laboratory Sciences and Infectious Diseases, Fondazione Policlinico Universitario A. Gemelli IRCCS, Rome, Italy); Serafina Valente and Alex Di Florio (Department of Cardiovascular Diseases, University of Siena, Siena, Italy); Marco Mandalà, Alessia Giorli, and Lorenzo Salerni (Otolaryngology Unit, University of Siena, Italy); Patrizia Zucchi and Pierpaolo Parravicini (Department of Internal Medicine, ASST Valtellina e Alto Lario, Sondrio, Italy); Elisabetta Menatti (Study Coordinator Oncologia Medica e Ufficio Flussi Sondrio, Italy); Tullio Trotta, Ferdinando Giannattasio, and Gabriella Coiro (First Aid Department, Luigi Curto Hospital, Polla, Salerno, Italy); Fabio Lena and Gianluca Lacerenza (Department of Pharmaceutical Medicine, Misericordia Hospital, Grosseto, Italy); Cristina Mussini (Infectious Diseases Clinics, University of Modena and Reggio Emilia); Luisa Tavecchia (U.O.C. Medicina, ASST Nord Milano, Ospedale Bassini, Cinisello Balsamo (MI), Italy); Lia Crotti, Gianfranco Parati, and Roberto Menè (IRCCS, Istituto Auxologico Italiano, Dipartimento di Cardiologia, Ospedale San Luca, Milano, Italy; Department of Medicine and Surgery, University of Milano-Bicocca, Milan, Italy; Istituto Auxologico Italiano, IRCCS, Center for Cardiac Arrhythmias of Genetic Origin, Milan, Italy; Istituto Auxologico Italiano, IRCCS, Laboratory of Cardiovascular Genetics, Milan, Italy; Member of the European Reference Network for Rare, Low Prevalence, and Complex Diseases of the Heart-ERN GUARD-Heart); Maurizio Sanarico (Independent Data Scientist, Milan, Italy); Marco Gori (University of Siena, DIISM-SAILAB, Siena, Italy; Maasai, I3S CNRS, Université Côte d’Azur, France); Francesco Raimondi and Alessandra Stella (Laboratorio di Biologia Bio@SNS, Scuola Normale Superiore, Pisa, Italy); Filippo Biscarini (CNR-Consiglio Nazionale delle Ricerche, Istituto di Biologia e Biotecnologia Agraria (IBBA), Milano, Italy); Tiziana Bachetti (Direzione Scientifica, Istituti Clinici Scientifici Maugeri IRCCS, Pavia, Italy); Maria Teresa La Rovere (Istituti Clinici Scientifici Maugeri IRCCS, Department of Cardiology, Institute of Montescano, Pavia, Italy); Maurizio Bussotti (Istituti Clinici Scientifici Maugeri IRCCS, Department of Cardiology, Institute of Milan, Italy); Serena Ludovisi (Fondazione IRCCS Ca’ Granda Ospedale Maggiore Policlinico, Milan, Italy); Katia Capitani (Core Research Laboratory, ISPRO, Florence, Italy); Simona Dei (Health Management, Azienda Usl Toscana sud est, Tuscany, Italy); Sabrina Ravaglia (IRCCS C. Mondino Foundation, Pavia, Italy); Annarita Giliberti, Giulia Gori, Rosangela Artuso, Elena Andreucci, Angelica Pagliazzi, and Erika Fiorentini (Medical Genetics Unit, Meyer Children’s University Hospital); Antonio Perrella and Francesco Bianchi (Department of Medicine, Pneumology Unit, Misericordia Hospital, Grosseto, Italy); Paola Bergomi, Emanuele Catena, and Riccardo Colombo (Department of Anesthesia and Intensive Care Unit, ASST Fatebenefratelli Sacco, Luigi Sacco Hospital, Polo Universitario, University of Milan, Milan); Sauro Luchi, Giovanna Morelli, Paola Petrocelli, Sarah Iacopini, and Sara Modica (Infectious Disease Unit, Hospital of Lucca, Italy); Silvia Baroni (Department of Diagnostic and Laboratory Medicine, Institute of Biochemistry and Clinical Biochemistry, Fondazione Policlinico Universitario A. Gemelli IRCCS, Catholic University of the Sacred Heart, Rome, Italy); Giulia Micheli (Clinic of Infectious Diseases, Catholic University of the Sacred Heart, Rome, Italy); Marco Falcone, Donato Urso, Giusy Tiseo, and Tommaso Matucci (Department of Clinical and Experimental Medicine, Infectious Diseases Unit, University of Pisa, Pisa, Italy); Davide Grassi and Claudio Ferri (Department of Clinical Medicine, Public Health, Life, and Environment Sciences, University of L’Aquila, Italy); Franco Marinangeli (Anesthesiology and Intensive Care, University of L’Aquila, L’Aquila, Italy); Francesco Brancati (Department of Life, Health, and Environmental Sciences, University of L’Aquila, L’Aquila, Italy; Human Functional Genomics Laboratory, IRCCS San Raffaele Roma, Rome, Italy); Antonella Vincenti, Valentina Borgo, Stefania Lombardi, and Mirco Lenzi (Infectious Disease Unit, Hospital of Massa, Italy); Massimo Antonio Di Pietro, Francesca Vichi, Benedetta Romanin, Letizia Attala, Cecilia Costa, Andrea Gabbuti, and Alessio Bellucci (Infectious Diseases Unit, Santa Maria Annunziata Hospital, USL Centro, Florence, Italy); Patrizia Casprini (Laboratory of Clinical Pathology and Immunoallergy, Florence–Prato, Italy); Cristoforo Pomara and Massimiliano Esposito (Department of Medical, Surgical, and Advanced Technologies “G.F. ingrassia,” University of Catania, Catania, Italy); Roberto Leoncini, Michele Cirianni, and Lucrezia Galasso (Laboratorio Patologia Clinica, Azienda Ospedaliero-Universitaria Senese); Marco Antonio Bellini (Ambulatory Chronic Polipathology of Siena, Department of Medicine, Surgery, and Neurosciences, University of Siena, Siena, Italy); Chiara Gabbi (Department of Biosciences and Nutrition, Karolinska Institutet, Stockholm, Sweden).

## References

[B1] Agosto A. Giudici P. (2020). A Poisson autoregressive model to understand covid-19 contagion dynamics. Risks 8, 77. 10.3390/risks8030077

[B2] Baldassarri M. Fava F. Fallerini C. Daga S. Benetti E. Zguro K. (2021a). Severe covid-19 in hospitalized carriers of single cftr pathogenic variants. J. personalized Med. 11, 558. 10.3390/jpm11060558 PMC8232773 34203982

[B3] Baldassarri M. Picchiotti N. Fava F. Fallerini C. Benetti E. Daga S. (2021b). Shorter androgen receptor polyq alleles protect against life-threatening covid-19 disease in european males. EBioMedicine 65, 103246. 10.1016/j.ebiom.2021.103246 33647767 PMC7908850

[B4] Ballow M. Haga C. L. (2021). Why do some people develop serious covid-19 disease after infection, while others only exhibit mild symptoms? J. Allergy Clin. Immunol. Pract. 9, 1442–1448. 10.1016/j.jaip.2021.01.012 33486141 PMC7825847

[B5] Benetti E. Giliberti A. Emiliozzi A. Valentino F. Bergantini L. Fallerini C. (2020a). Clinical and molecular characterization of covid-19 hospitalized patients. Plos one 15, e0242534. 10.1371/journal.pone.0242534 33206719 PMC7673557

[B6] Benetti E. Tita R. Spiga O. Ciolfi A. Birolo G. Bruselles A. (2020b). Ace2 gene variants may underlie interindividual variability and susceptibility to covid-19 in the Italian population. Eur. J. Hum. Genet. 28, 1602–1614. 10.1038/s41431-020-0691-z 32681121 PMC7366459

[B10] Bialek S. Boundy E. Bowen V. Chow N. Cohn A. Dowling N. CDC COVID-19 Response Team (2020). Severe outcomes among patients with coronavirus disease 2019 (COVID-19) - United States, february 12-march 16, 2020. Morb. Mortal. Wkly. Rep. 69, 343–346. 10.15585/mmwr.mm6912e2 PMC7725513 32214079

[B7] Boyle E. A. Li Y. I. Pritchard J. K. (2017). An expanded view of complex traits: from polygenic to omnigenic. Cell. 169, 1177–1186. 10.1016/j.cell.2017.05.038 28622505 PMC5536862

[B8] Castorina P. Iorio A. Lanteri D. (2020). Data analysis on coronavirus spreading by macroscopic growth laws. Int. J. Mod. Phys. C 31, 2050103. 10.1142/s012918312050103x

[B9] Caux J.-S. (2016). The quench action. J. Stat. Mech. Theory Exp. 2016, 064006. 10.1088/1742-5468/2016/06/064006

[B11] Chen Y. Liu Q. Guo D. (2020). Emerging coronaviruses: genome structure, replication, and pathogenesis. J. Med. virology 92, 2249–2423. 10.1002/jmv.26234 32881013 PMC7435528

[B12] COVID-19 Host Genetics Initiative, Karjalainen J. Liao R. G. Neale B. M. Daly M. Ganna A. (2021). Mapping the human genetic architecture of covid-19. Nature 600, 472–477. 10.1038/s41586-021-03767-x 34237774 PMC8674144

[B13] Croci S. Venneri M. A. Mantovani S. Fallerini C. Benetti E. Picchiotti N. (2022). The polymorphism l412f in tlr3 inhibits autophagy and is a marker of severe covid-19 in males. Autophagy 18, 1662–1672. 10.1080/15548627.2021.1995152 34964709 PMC9298458

[B14] Daga S. Fallerini C. Baldassarri M. Fava F. Valentino F. Doddato G. (2021). Employing a systematic approach to biobanking and analyzing clinical and genetic data for advancing covid-19 research. Eur. J. Hum. Genet. 29, 745–759. 10.1038/s41431-020-00793-7 33456056 PMC7811682

[B15] Dong E. Du H. Gardner L. (2020). An interactive web-based dashboard to track covid-19 in real time. Lancet Infect. Dis. 20, 533–534. 10.1016/S1473-3099(20)30120-1 32087114 PMC7159018

[B16] Fallerini C. Daga S. Benetti E. Picchiotti N. Zguro K. Catapano F. (2021a). Selp asp603asn and severe thrombosis in covid-19 males. J. Hematol. Oncol. 14, 123–124. 10.1186/s13045-021-01136-9 34399825 PMC8365289

[B17] Fallerini C. Daga S. Mantovani S. Benetti E. Picchiotti N. Francisci D. (2021b). Association of toll-like receptor 7 variants with life-threatening covid-19 disease in males: findings from a nested case-control study. elife 10, e67569. 10.7554/eLife.67569 33650967 PMC7987337

[B18] Fallerini C. Picchiotti N. Baldassarri M. Zguro K. Daga S. Fava F. (2022). Common, low-frequency, rare, and ultra-rare coding variants contribute to covid-19 severity. Hum. Genet. 141, 147–173. 10.1007/s00439-021-02397-7 34889978 PMC8661833

[B19] Fanelli D. Piazza F. (2020). Analysis and forecast of covid-19 spreading in China, Italy and France. Chaos, Solit. Fractals 134, 109761. 10.1016/j.chaos.2020.109761 PMC7156225 32308258

[B20] Fenga L. (2021). Covid-19: an automatic, semiparametric estimation method for the population infected in Italy. PeerJ 9, e10819. 10.7717/peerj.10819 33717677 PMC7937344

[B21] Gad A. F. (2021). Pygad: an intuitive genetic algorithm python library. *arXiv preprint arXiv:2106.06158* .

[B22] Gebhard C. Regitz-Zagrosek V. Neuhauser H. K. Morgan R. Klein S. L. (2020). Impact of sex and gender on covid-19 outcomes in europe. Biol. sex Differ. 11, 29–13. 10.1186/s13293-020-00304-9 32450906 PMC7247289

[B23] Hütter G. Blüthgen C. Neumann M. Reinwald M. Nowak D. Klüter H. (2013). Coregulation of hiv-1 dependency factors in individuals heterozygous to the ccr5-delta32 deletion. AIDS Res. Ther. 10, 26–28. 10.1186/1742-6405-10-26 24245779 PMC3834523

[B24] Jin J.-M. Bai P. He W. Wu F. Liu X.-F. Han D.-M. (2020). Gender differences in patients with covid-19: focus on severity and mortality. Front. public health 152. 10.3389/fpubh.2020.00152 PMC7201103 32411652

[B25] Kosmicki J. A. Horowitz J. E. Banerjee N. Lanche R. Marcketta A. Maxwell E. (2021). Pan-ancestry exome-wide association analyses of covid-19 outcomes in 586,157 individuals. Am. J. Hum. Genet. 108, 1350–1355. 10.1016/j.ajhg.2021.05.017 34115965 PMC8173480

[B26] Kousathanas A. Pairo-Castineira E. Rawlik K. Stuckey A. Odhams C. A. Walker S. (2021). Whole genome sequencing identifies multiple loci for critical illness caused by covid-19. medRxiv.

[B27] Kramer O. Kramer O. (2016). Scikit-learn. Mach. Learn. Evol. strategies, 45–53. 10.1007/978-3-319-33383-0_5

[B28] Lai A. Bergna A. Acciarri C. Galli M. Zehender G. (2020). Early phylogenetic estimate of the effective reproduction number of sars-cov-2. J. Med. virology 92, 675–679. 10.1002/jmv.25723 32096566 PMC7228357

[B29] Lanteri D. Carco D. Castorina P. (2020). How macroscopic laws describe complex dynamics: asymptomatic population and covid-19 spreading. Int. J. Mod. Phys. C 31, 2050112. 10.1142/s0129183120501120

[B30] Madabhavi I. Sarkar M. Kadakol N. (2020). Covid-19: a review. Monaldi Archives Chest Dis. 90. 10.4081/monaldi.2020.1298 32498503

[B31] Mantovani S. Daga S. Fallerini C. Baldassarri M. Benetti E. Picchiotti N. (2022). Rare variants in toll-like receptor 7 results in functional impairment and downregulation of cytokine-mediated signaling in covid-19 patients. Genes. & Immun. 23, 51–56. 10.1038/s41435-021-00157-1 PMC8703210 34952932

[B32] Marouli E. Graff M. Medina-Gomez C. Lo K. S. Wood A. R. Kjaer T. R. (2017). Rare and low-frequency coding variants alter human adult height. Nature 542, 186–190. 10.1038/nature21039 28146470 PMC5302847

[B33] Martelloni G. Martelloni G. (2020a). Analysis of the evolution of the sars-cov-2 in Italy, the role of the asymptomatics and the success of logistic model. Chaos, Solit. Fractals 140, 110150. 10.1016/j.chaos.2020.110150 PMC7386499 32834638

[B34] Martelloni G. Martelloni G. (2020b). Modelling the downhill of the sars-cov-2 in Italy and a universal forecast of the epidemic in the world. Chaos, Solit. Fractals 139, 110064. 10.1016/j.chaos.2020.110064 PMC7328650 32834614

[B35] Monticelli M. Hay Mele B. Benetti E. Fallerini C. Baldassarri M. Furini S. (2021). Protective role of a tmprss2 variant on severe covid-19 outcome in young males and elderly women. Genes. 12, 596. 10.3390/genes12040596 33921689 PMC8073081

[B36] Mukherjee S. Pahan K. (2021). Is covid-19 gender-sensitive? J. Neuroimmune Pharmacol. 16, 38–47. 10.1007/s11481-020-09974-z 33405098 PMC7786186

[B37] O’Brien J. Du K. Y. Peng C. (2020). Incidence, clinical features, and outcomes of covid-19 in Canada: impact of sex and age. J. ovarian Res. 13, 1–12. 10.1186/s13048-020-00734-4 PMC7684854 33234144

[B38] Pairo-Castineira E. Clohisey S. Klaric L. Bretherick A. D. Rawlik K. Pasko D. (2021). Genetic mechanisms of critical illness in covid-19. Nature 591, 92–98. 10.1038/s41586-020-03065-y 33307546

[B39] Pedregosa F. Varoquaux G. Gramfort A. Michel V. Thirion B. Grisel O. (2011). Scikit-learn: machine learning in python. J. Mach. Learn. Res. 12, 2825–2830. 10.5555/1953048.2078195

[B40] Picchiotti N. Benetti E. Fallerini C. Daga S. Baldassarri M. Fava F. (2021). Post-mendelian genetic model in covid-19. Cardiol. Cardiovasc. Med. 5 (6), 673–694. 10.26502/fccm.92920232

[B41] Severe Covid-19 GWAS Group, Ellinghaus D. Degenhardt F. Bujanda L. Buti M. Albillos A. (2020). Genomewide association study of severe covid-19 with respiratory failure. N. Engl. J. Med. 383, 1522–1534. 10.1056/NEJMoa2020283 32558485 PMC7315890

[B42] Stehman S. V. (1997). Selecting and interpreting measures of thematic classification accuracy. Remote Sens. Environ. 62, 77–89. 10.1016/s0034-4257(97)00083-7

[B43] Wu C. Chen X. Cai Y. Zhou X. Xu S. Huang H. (2020). Risk factors associated with acute respiratory distress syndrome and death in patients with coronavirus disease 2019 pneumonia in wuhan, China. JAMA Intern. Med. 180, 934–943. 10.1001/jamainternmed.2020.0994 32167524 PMC7070509

