# Supplementary material for: Host genetics and COVID-19 severity: increasing the accuracy of latest severity scores by Boolean quantum features
Source: Front Genet. 2024 May 22;15:1362469. doi: 10.3389/fgene.2024.1362469 (PMC11150643; doi:10.3389/fgene.2024.1362469)
Supplement: Supplementary file 2 [file DataSheet1.PDF]

Host genetics and COVID-19 severity: increasing the accuracy of latest severity scores by Boolean quantum features - Supplementary Information

Gabriele Martelloni[a], Alessio Turchi[b], Chiara Fallerini[a,c], Andrea Degl'Innocenti[a], Margherita Baldassarri[a], GEN-COVID Multicenter study[d], Simona Olmi[e], Simone Furini[f], Alessandra Renieri[a,c]

[a] *Medical Genetics Unit, Azienda Ospedaliera Universitaria Senese, viale Bracci 2, 53100 Siena, Italy*
[b] *INAF Osservatorio Astrofisico di Arcetri, Largo Enrico Fermi 5, I-50125 Firenze, Siena*
[c] *Med Biotech Hub andCompetence Center, Department of Medical Biotechnologies, University of Siena, Viale Mario Bracci, 16, 53100 Siena, Italy*
[d] *Lists of authors and their affiliations appear at the end of the main text*
[e] *CNR-Consiglio Nazionale delle Ricerche - Istituto dei Sistemi Complessi, via Madonna del Piano 10, 50019 Sesto Fiorentino, Italy*
[f] *Department of Electrical, Electronic and Information Engineering "Guglielmo Marconi", University of Bologna, via dell'Università 50, Cesena (FC), 47521, Italy*

Key words: COVID19, Host Genetics, Integrated PolyGenic Score, Genetic Algorithm, Logistic Regression, Genetic science modelling.

1. Some simple example of $\mathbf{IPGS}^{1}_{ph}$

Example 1. Let us consider the simplest possible case:

- patient with only one organ involved, the heart for example;
- patient with only one mild variant involved (rare).

We will therefore have

$$\mathrm{IPGS}^{1}_{ph} = F_r p_i O_{ij}^{\mathrm{hearth}} v_j^m, \tag{1}$$

where

$$p_i = \begin{pmatrix} 1 \\ 0 \end{pmatrix}, \tag{2}$$

$$v_j^m = \begin{pmatrix} 1 \\ 0 \end{pmatrix}, \tag{3}$$

$$O_{ij}^{\mathrm{hearth}} = \begin{pmatrix} \alpha_h & 0 \\ 0 & 0 \end{pmatrix}. \tag{4}$$

Email addresses: g.martelloni@student.unisi.it (Gabriele Martelloni), simona.olmi@fi.isc.cnr.it (Simona Olmi)

The result reads

$$\mathrm{IPGS}^1_{ph} = F_r \alpha_h, \tag{5}$$

obviously in the limit that the organs do not have a coefficient and therefore $\alpha_c = 1$, we go to the limit of the old $IPGS = F_r$, that is with $n_r^m = 1$ and all others $n = 0$.

Example 2. Let's consider another simple case:

- patient with two organs involved, the heart and kidney for example;
- patient with two variants involved, a mild rare and a severe ultrarare.

Then we obtain

$$\mathrm{IPGS}^1_{ph} = F_r p_i O_{ij} v_j^m + F_{ur} p_i O_{ij} v_j^s, \tag{6}$$

where

$$p_i = \begin{pmatrix} 1 \\ 0 \\ 1 \\ 0 \end{pmatrix}, \tag{7}$$

$$v_j^m = \begin{pmatrix} 1 \\ 0 \\ 1 \\ 0 \end{pmatrix}, \tag{8}$$

$$v_j^s = \begin{pmatrix} 1 \\ 0 \\ 1 \\ 0 \end{pmatrix}, \tag{9}$$

$$O_{ij} = \begin{pmatrix} \alpha_h & 0 & 0 & 0 \\ 0 & 0 & 0 & 0 \\ 0 & 0 & \alpha_k & 0 \\ 0 & 0 & 0 & 0 \end{pmatrix}, \tag{10}$$

whose result will be

$$\mathrm{IPGS}^1_{ph} = F_r(\alpha_h + \alpha_k) + F_{ur}(\alpha_h + \alpha_k). \tag{11}$$

We begin to notice the first important differences with this simple example, the vector that represents the variants, is no longer a two-component vector, but a vector of 4 so that it can take count more organs involved, in fact as long as the coefficients of the organs go to 1, the result becomes

$$\mathrm{IPGS}^1_{ph} = 2F_r + 2F_{ur}, \tag{12}$$

which does not coincide with the old IPGS

$$\mathrm{IPGS}^1_{ph} = F_r - F_{ur}, \tag{13}$$

we begin to weigh variants involved in several organs in a different way.

Example 3. Finally, let's consider another case:

- patient with only one organ involved, the heart and with a negative history of the kidney for example;
- patient with two variants involved, a mild rare and a severe ultrarare.

So we have

$$\mathrm{IPGS}^1_{ph} = F_r p_i O_{ij} v^m_j + F_{ur} p_i O_{ij} v^s_j, \tag{14}$$

where

$$p_i = \begin{pmatrix} 1 \\ 0 \\ 0 \\ 1 \end{pmatrix}, \tag{15}$$

$$v^m_j = \begin{pmatrix} 1 \\ 0 \\ 0 \\ 1 \end{pmatrix}, \tag{16}$$

$$v^s_j = \begin{pmatrix} 1 \\ 0 \\ 0 \\ 1 \end{pmatrix}, \tag{17}$$

$$O_{ij} = \begin{pmatrix} \alpha_h & 0 & 0 & 0 \\ 0 & 0 & 0 & 0 \\ 0 & 0 & \alpha_k & 0 \\ 0 & 0 & 0 & 0 \end{pmatrix}, \tag{18}$$

whose result will be

$$\mathrm{IPGS}^1_{ph} = F_r \alpha_h + F_{ur} \alpha_h. \tag{19}$$

With this example we observe how the vector of patients in the absence of an organ involved by the vector $\begin{pmatrix} 1 \\ 0 \end{pmatrix}$, it is transformed into the vector $\begin{pmatrix} 0 \\ 1 \end{pmatrix}$ for having null scalar product on that precise organ.

2. Quantum Mechanics and time evolution

Quantum Mechanics (QM) was born in the early 1900's to explain some behaviors of light and electrons at the energies of the atomic scale, in particular the fact that light behaved for Classical Mechanics (CM) as a wave and the electron as a particle was not enough to explain some phenomena such as diffraction on a small slit and the stability of the atom. For quantum mechanics, every physical observable in nature has a double behavior, both as a wave and as a particle. This idea becomes a complete shift paradigm also in the mathematical representation of the physical observables themselves and also of their meaning:

- in CM the position and time, in which we described our physical system, have a deterministic interpretation;
- in QM the concept of position and time is replaced by the concept of wave function ($\psi(x,t)$ as a function or $|\psi(x,t)>$ as a vector), whose modulus represents the probability of finding our physical system at that precise time and at those coordinates;
- the mathematical concept of physical observable changes: in CM a physical observable is a function of space and time, for example the electric field of a point charge $\vec{E}(x,t)$, in QM an observable is an operator (technically a matrix), whose expectation value on the wave function will be its measure in that state:

$$< \psi(x,t)|\mathrm{O}|\psi(x,t) >=< \psi(x,t)|\mathrm{A}|\psi(x,t) >= \mathrm{A} < \psi(x,t)|\psi(x,t) >= \mathrm{A}, \tag{20}$$

  where A is the eigenvalue of the observable O on the normalized wave function $\psi(x,t)$, basically It is a dot product of the row-matrix-column type;
- in CM the temporal evolution of a particle is studied through Newton's second law

$$\vec{F} = m\vec{a} = m\ddot{\vec{x}}, \tag{21}$$

  through the second derivatives of the vector position respect to time, this often implies a non-linearity of the problem;
- instead in QM the problem becomes linear in the time derivative which is represented by the Hamiltonian operator or by the energy of the system (kinetic energy + potential of the system), this is the well-known Schrödinger's equation:

$$\imath\hbar\frac{\partial}{\partial t}\psi(x,t) = \mathrm{H}\psi(x,t) = (\frac{p^2}{2m} + U(x,t))\psi(x,t). \tag{22}$$

- with respect to CM the energy is no longer a continuum of values but it is discretized in energy quanta $E = n\hbar\omega$ where n takes values in natural number ensemble and ω is the frequency of the single quantum of energy, this is the other great revolution of QM, the concept of discretization of nature. Finally, solving the Schrödinger equation we have that the time evolution operator $U(t,t_0)$ becomes

$$U(t,t_0)|\psi(x,t) >= e^{-i\frac{H(t-t_0)}{\hbar}}|\psi(x,t) >= e^{-i\frac{E(t-t_0)}{\hbar}}|\psi(x,t) >, \tag{23}$$

  where E is the eigenvalue of the energy of that state.
